# Pediatric Headache in Primary Care and Emergency Departments: Consensus with RAND/UCLA Method

**DOI:** 10.3390/life12020142

**Published:** 2022-01-19

**Authors:** Giovanni Prezioso, Agnese Suppiej, Valentina Alberghini, Patrizia Bergonzini, Maria Elena Capra, Ilaria Corsini, Alessandro De Fanti, Elisa Fiumana, Martina Fornaro, Lucia Marangio, Paolo Ricciardelli, Laura Serra, Duccio Maria Cordelli, Susanna Esposito, the Emilia-Romagna Headache Study Group

**Affiliations:** 1 Clinic of Pediatrics, Department of Medicine and Surgery, University Hospital of Parma, 43126 Parma, Italy; gprezioso@hotmail.it; 2 Unit of Pediatrics, Sant’Anna University Hospital, 44124 Ferrara, Italy; agnese.suppiej@unife.it (A.S.); e.fiumana@ospfe.it (E.F.); 3 Unit of Pediatrics, Maggiore Hospital, 40133 Bologna, Italy; valentina.alberghini@ausl.bologna.it; 4 Unit of Pediatrics, University Hospital of Modena, 41125 Modena, Italy; bergonzini.patriza@aou.mo.it; 5 Unit of Pediatrics and Neonatology, G. di Saliceto Hospital, 29121 Piacenza, Italy; m.capra@ausl.pc.it; 6 Emergency Pediatrics, IRCCS Azienda Ospedaliera—Universitaria di Bologna, 40138 Bologna, Italy; ilariacorsini@hotmail.com; 7 Unit of Pediatrics, IRCCS-AUSL Reggio Emilia, 42100 Reggio Emilia, Italy; Alessandro.DeFanti@ausl.re.it; 8 Unit of Neonatology and Pediatrics, Macerata Hospital, 62100 Macerata, Italy; martina.fornaro@sanita.marche.it; 9 Unit of Pediatrics, Forlì Hospital, AUSL Romagna, 47121 Forlì, Italy; lucia.marangio@auslromagna.it; 10 Unit of Pediatrics, Ravenna Hospital, AUSL Romagna, 48100 Ravenna, Italy; paolo.ricciardelli@auslromagna.it; 11 Unit of Pediatrics, AUSL Imola, 40026 Imola, Italy; l.serra@ausl.imola.bo.it; 12 Unit of Child Neuropsychiatry, IRCCS Azienda Ospedaliera—Universitaria di Bologna, 40138 Bologna, Italy; ducciomaria.cordelli@unibo.it

**Keywords:** headache, migraine, red flags, RAND/UCLA appropriateness method, pediatric headache, acute therapy, prophylaxis

## Abstract

Headache is the most frequent neurological symptom in childhood and the main reason for admission to pediatric emergency departments. The aim of this consensus document is to define a shared clinical pathway between primary care pediatricians (PCP) and hospitals for the management of children presenting with headache. For the purposes of the study, a group of hospital pediatricians and a group of PCP from the Emilia Romagna’s health districts were selected to achieve consensus using the RAND/UCLA appropriateness method. Thirty-nine clinical scenarios were developed: for each scenario, participants were asked to rank the appropriateness of each option from 1 to 9. Agreement was reached if ≥75% of participants ranked within the same range of appropriateness. The answers, results, and discussion helped to define the appropriateness of procedures with a low level of evidence regarding different steps of the diagnostic-therapeutic process: primary care evaluation, emergency department evaluation, hospital admission, acute therapy, prophylaxis, and follow-up. The RAND proved to be a valid method to value appropriateness of procedures and define a diagnostic-therapeutic pathway suitable to the local reality in the management of pediatric headache. From our results, some useful recommendations were developed for optimizing the healthcare professionals’ network among primary care services and hospitals.

## 1. Introduction

Headache is the most frequent neurological symptom in childhood, with a general prevalence in the pediatric population estimated at about 58.4% according to a recent study [1,2,3]. Headache also represents the main reason for admission to pediatric emergency departments with a rate ranging from 0.6 to 1.3% of total cases [4,5,6], most of them being related to upper airway infections [4]. Recurring headache prevalence varies with age, from 4.5% in children aged 4 to 6 years to 27.4% in adolescents aged 16 to 18 years [7].

The aim of this consensus document is to define a shared clinical pathway between primary care pediatricians (PCPs) and hospitals for the management of children presenting with headache. In consideration of the multiple etiologies, such as the high inappropriate emergency department (ED) access rate, as well as the differences between inpatient and outpatient management, an extensive collaboration among the involved healthcare professionals is desirable. This shared diagnostic approach will give useful recommendations to primary healthcare and the hospital professionals involved, thus improving the standard of care and quality of life of these patients.

## 2. Methods

The consensus document was realized using the Research and Development Corporation (RAND) and the University of California—Los Angeles (UCLA) appropriateness method. The RAND/UCLA method consists of an appropriateness evaluation of diagnostic and therapeutic procedures with sub-optimal scientific evidence by a panel of experts [8]. According to the RAND method, a procedure is defined as “appropriate” if the expected benefits outweigh the expected negative consequences, with a wide margin that justifies it, regardless of the costs. On the contrary, a procedure whose expected risks outweigh the expected benefits is considered as “inappropriate”. According to the RAND definition, an expert who makes a judgment on appropriateness/inappropriateness must consider the clinical benefits and not be influenced by economic considerations. Therefore, appropriateness accounts for the evaluation of the risk/benefit ratio of a list of diagnostic, management, and therapeutic procedures [9].

A group of selected experts anonymously answered a questionnaire, whose results are discussed in a collegial meeting; after that, a second survey was performed to reduce eventual disagreement [10]. Every answer was then classified as “appropriate”, “inappropriate”, or “uncertain”. The aims of the questionnaire were to define: (1) approach in primary care; (2) criteria for ED admission and hospitalization of children with headache; (3) a diagnostic approach for hospitalized children; (4) the best evidence-based acute and prophylactic treatment of headache disorders and migraine in children.

In this study, the questionnaire (Material S1) was organized in 39 clinical scenarios developed by the project coordinators together with the Pediatrics Departments Directors of Emilia-Romagna and the Pediatric Neuropsychiatry Department Director of Bologna University. The clinical scenarios were proposed after a careful revision of current scientific literature, including original research works, reviews and systematic reviews, meta-analysis, recommendations and guidelines, selected using MEDLINE. For every scenario, a review based on literature revision was provided to the experts panel after the survey. After a discussion about the results and implementation, 25 recommendations were defined.

The panel of experts consisted of a group of hospital pediatricians and a group of primary care pediatricians from the Emilia Romagna’s health districts, in proportion to their pediatric population size according to 2018 Italian National Statistics Institute (ISTAT) data.

Expert judgment was expressed on a scale of 1–9, where “1” was considered definitely inappropriate, “5” was considered uncertain, an “9” was considered definitely appropriate. Intermediate values corresponded to different modulations of the judgment of inappropriateness (“2” and “3”), uncertainty (from “4” to “6”), and appropriateness (“7” and “8”), respectively. In evaluating each indication, each expert referred both to their own experience and clinical judgment and to the available scientific evidence. Free space was provided for any annotations or comments.

The kick-off meeting was set at 22 September 2021 via Teams. The first round of the questionnaire was blind to other panel members. The experts responded through an online survey application “Google forms” with a one-month deadline. Round 1 responses were collected and sent to an independent statistician for analysis. Results of the survey were discussed in the kick-off meeting, during which the collective ranking of scenarios and indications was shown. Clarifications, adaptations, and refinements of the indications and appropriateness ratings were made; thus, participants were asked to re-rank the scenarios in a second round during the following four weeks. The results of the second round, presented on 28 October 2021, were considered definitive.

Questionnaire results for each scenario were reported as frequencies and medians. Median and discordance were classified into three levels of appropriateness (appropriate: between ‘7’ and ‘9’, without disagreement; uncertain: between ‘4’ and ‘6’ or any median with disagreement; inappropriate: between ‘l’ and ‘3’ with agreement) (Table 1).

Agreement was considered to be reached in the case of at least 75% of respondents ranking within the same range of appropriateness. The data analysis was performed with the STATA^®^ Statistical Software (Release 11 College Station, TX, USA). The mean value with a 95% confidence interval was calculated. Microsoft Excel^®^ was used for graphic data processing and presentation.

Results were resumed in bar and pie charts, as shown in the Material S2.

## 3. Results

Thirty-six hospital pediatricians and PCPs responded to the first survey, while 24 panelists participated in the second survey.

Supplement Material S1 shows the administered questionnaire and Supplement Material S2 includes the bar and pie charts derived from panelists’ answers to the first and second surveys.

The recommendations developed by the coordination group are presented below (summarized in Table 2) and divided into six parts representing the six phases of the diagnostic-therapeutic pathway for headache management: patient evaluation by the PCP, ED evaluation, hospital admission, acute therapy, prophylaxis and follow-up.

### 3.1. Primary Care Pediatrician Evaluation

#### 3.1.1. Recommendation 1 (Scenario 1)

The following represent anamnestic red flags for secondary headaches:

- Age < 3 years
- Nocturnal or awakening headache
- Vomiting at night or in early morning
- Onset after physical effort, cough, Valsalva maneuver
- Cognitive decline or personality changes
- Altered state of consciousness
- History of seizures
- Recent onset (<2 months)
- Explosive onset
- Rapid worsening
- Fixed unilateral headache
- Association with projectile vomit, fever, general malaise
- Slow-down of weight for height development
- Change in headache pattern in children with known primary headache.

According to data reported in the literature, the assessment of pediatric patients with headache is based as a first step on an adequate medical history, focused on searching for the so-called “red flags”, warning signs suggestive of secondary headache. Among these “red flags”, some are considered at high risk, as shown in Table 2 and Table 3 [11,12]. In cases with recent onset of headache (<6 months), nocturnal and/or early-morning presentation with or without projectile vomiting, positive neurological examination, or an altered state of consciousness or personality, the probability of an association to an intracranial expansive process increases by approximately 4% [13,14].

Useful information also comes from the age at onset and presentation patterns (Table 4) [15].

Statistically, the first episode of severe acute headache is more frequently associated with fever during upper respiratory tract infections or migraines in pre-school age (2–5 years) [11]. However, if the child’s age is below 5 years this should be considered a red flag, as the highest prevalence of primary forms occur in school age (30–50%) and adolescence (50–80%) [7].

Sudden onset of a headache of severe and progressive intensity (e.g., “thunderclap” headache or “worst headache episode of my life”) may also be indicative of vascular events, such as intracranial hemorrhage, carotid dissection, or venous sinus thrombosis [16,17].

A chronic progressive headache pattern should be carefully evaluated because it may be related to a gradual increase in intracranial pressure, resulting from brain tumors, hydrocephalus, brain abscesses, vascular malformations, and vascular hemorrhage. More typical primary headaches have a chronic non-progressive pattern [18]. However, significant and worsening variations in temporal patterns may imply the occurrence of a secondary headache on a previous primary form [19].

A family or personal history of epilepsy, in a recurrent acute headache pattern, may address secondary forms of headache with epileptic variants [18]. Indeed, there have been specific types of epilepsy occurring simultaneously or immediately after a migraine aura (migralepsy) and forms of headache that are the only manifestation of ictal electrical activity (epileptic headache), but authors still have divergent opinions about definitions [20].

Headache that worsens with sneezing or coughing, triggered by Valsalva maneuver, exertion, and physical activity may be suggestive of intracranial hypertension [13]. In contrast, exacerbation with a transition from clino- to orthostatism may suggest intracranial hypotension [13].

The presence of symptoms temporally associated with the headache episode is further support to differential diagnosis. The classic neurological manifestations of migraine auras, like paresthesias, fortification spectra, and scintillating scotomas are less common in prepubertal children. Visual disturbances preceding or associated with the attack are the most frequent symptoms in migraines, but it is important to carefully distinguish any symptoms suggestive of occipital epilepsy (see Recommendation 2). Symptoms such as nausea, vomiting, and myalgias are frequently associated to primary headache in pediatric age [21]. The presence of vomiting and nocturnal awakenings is generally associated with an increased risk of secondary forms, although some retrospective studies have not shown correlations [22,23]. Hypo/hyperactivity, fatigue, transient speech changes, neck stiffness and/or pain are aspecific warning signs involving the brainstem but may represent the neurological manifestations of basilar origin migraine, although very rare in pediatric age.

Another salient aspect of the clinical history is headache localization [19,21,22]. An exclusively unilateral headache in a pediatric patient, especially when associated with other red flags, may be suggestive of secondary forms, and this is because migraines in children are often bilateral, frontal or temporal, whereas tension-type headaches have a more diffuse localization [23]. Endocrinological changes, such as growth retardation, precocious puberty, galactorrhoea, diabetes insipidus, hypo-hyperthyroidism, and obesity raise suspicions of chiasmatic, hypothalamic or pituitary lesions [24].

**Recommendation** **1.** *In the case of acute headache in a pediatric patient, the PCP should identify the red flags that require urgent diagnostic and therapeutic procedures by an adequate history collection, in order to distinguish primary from secondary forms.*

##### First and Second Survey Results Comparison

Most of the statements of Scenario 1 were rated with agreement at the first round. Only a recent onset of headache (<2 months) and the slow-down of the weight for height development obtained agreement for appropriateness as red flags after the second survey.

#### 3.1.2. Recommendation 2 (Scenario 2)

The following are red flags for secondary headaches on physical examination:

- Macrocephaly in infants
- Meningeal signs
- Neurocutaneous markers
- Papilledema
- Abnormal eye movements, diplopia or nystagmus
- Asymmetry of strength or sensation
- Disturbance of gait or balance
- Asymmetry of osteotendinous reflexes

The general and neurological examination allows for the identification of several signs and symptoms suggestive of secondary headache (Table 5 and Table 6).

The finding of an elevated head circumference or disharmonic head growth before sutures and anterior fontanel closure could be a sign of chronic progressive increase in intracranial pressure [18]. In these cases, papilledema on ocular fundus examination (OF) may be absent and the child may show only non-specific signs, such as irritability, vomiting, and crying.

A headache episode with fever and typical features, such as drowsiness, irritability, nuchal rigidity and positivity of some maneuvers, such as Kernig’s and Brudzinski’s meningeal signs, is indicative of a severe intracranial infectious process [18].

On the other hand, the presence of skin dyschromia may be suggestive of neurocutaneous diseases. The most common are tuberous sclerosis, that may be associated with brain masses (cortical/subcortical glioneuronal tubers, subependymal glial nodules or giant cell astrocytomas), and neurofibromatosis type 1, associated with gliomas, hydrocephalus due to congenital stenosis of Silvio’s aqueduct or, more rarely, Moyamoya vasculopathy [11,18].

Abnormalities on neurological examination, such as alterations in mental state, visual disturbances (reduced vision, narrowing of the visual field, altered extrinsic ocular motility with nystagmus and/or diplopia, visual field alterations), lateralizing signs or deficits of strength and/or sensitivity of cranial or peripheral nerves, alterations in walking and balance, or alterations in osteotendinous reflexes should be considered high-risk objective red flags, and if associated with anamnestic red flags for expansive or vascular brain lesions (see Recommendation 1), urgent neuroradiological investigations are indicated [12,25]. However, focal neurological manifestations with or without visual disturbances and paresthesias may be due to a migraine with aura [14]. Differential diagnosis can be difficult, but the temporal pattern may be of help: focal neurological symptoms of aura develop over several minutes and last less than 60 min, whereas a more rapid onset or longer duration of symptoms makes a secondary etiology more likely. Refer to the third edition of the International Classification of Headache Disorder (ICHD-III) for detailed criteria [3].

Within the neurological examination, the OF is pivotal, since it allows to rule out papilledema, and therefore, potential intracranial hypertension. However, additional skills and an adequate clinical experience are required for a pediatrician to perform this exam, also considering the peculiarities and compliance of a pediatric patient. Furthermore, instruments available to a non-specialist do not always allow correct visualization of the fundus; therefore, if intracranial hypertension is suspected and the optic papilla visualization is suboptimal, ophthalmologist consultations for OF with cycloplegia is appropriate [22,26].

**Recommendation** **2.** *Assessment of vital signs, general physical examination, and a complete neurological examination should always be performed in a child with headache, as this can identify warning signs suggestive of secondary headache. In particular, the presence of high-risk red flags suggestive of severe conditions such as infectious processes, vascular lesions or intracranial expansions should be excluded. The neurological examination should include: assessment of level of consciousness, meningeal signs, visual, gait and co-ordination disturbances, speech and hearing disorders, focal neurological deficits such as localized strength or sensory deficits, and cranial nerve deficits. The OF is a useful non-invasive examination that a pediatrician can perform if sufficiently experienced. In doubtful cases, an ophthalmologist evaluation for fundoscopy with pupil dilation is recommended*.

##### First and Second Survey Results Comparison

Except for macrocephaly, the answers of Scenario 2 reached agreement after the first survey. Correction in the discussion phase for Answer B from macrocephaly to macrocephaly in toddlers led to an increase in the appropriateness rating, from 61.1% to 91.7%.

#### 3.1.3. Recommendation 3 (Scenarios 3 and 4)

ED referral is indicated in a patient with headache and red flags in cases of:

- Sudden onset of severe headache
- Headache exacerbated by lying down or coughing
- Focal neurological signs
- Dysautonomic signs (nausea, vomiting, paleness, sweating, etc.) preceding the headache
- Headache with temporal localization
- Precocious puberty
- Headache with occipital localization
- Chronic progressive headache

It is recommended to send the child to the ED in the following cases:

- A 14-year-old patient with a history of tension-type headache for two years with a sudden onset of severe headache
- A 15-year-old patient with a history of migraine and an episode partially responsive to paracetamol, associated with nausea and vomiting not present at night or in early morning
- An 8-year-old patient with headache and fever for two days with upper respiratory tract infection
- A 9-year-old patient with headache and high fever for four days with drowsiness and photophobia
- An 11-year-old patient with headache triggered or worsen by lying down or coughing
- A 14-year-old female patient with her third headache episode in five months preceded by aspecific visual disturbances of 15–30 min and strength deficit of the left arm
- A 17-year-old patient with known migraine, presenting with an episode lasting longer than 72 h and unresponsive to ibuprofen and paracetamol
- A 15-year-old patient with migraine without aura lasting more than 48 h and unresponsive to paracetamol

As explained in Recommendation 1, in the case of anamnestic or clinical red flags suggestive of potentially life-threatening conditions, the patient should be immediately referred to an ED [4,11,13,18].

In a pediatric patient, particularly of prepubertal age, headache with unilateral localization may be a warning sign suggestive of a secondary form [23]. However, the global clinical picture needs to be considered, as it has been shown that in pediatric populations, a unilateral localization on its own, particularly if occipital, does not correlate with an increased risk of intracranial structural lesions. Only in the case of a positive neurological exam (typically, walking, motor coordination, and/or balance anomalies) and/or OF indicative of papilledema are further diagnostic investigations needed [4,27].

Similarly, a history of headache in association with precocious puberty or other endocrinological alterations needs further investigation, as that is potentially related to a lesion of the sellar region [24].

**Figure 1:**
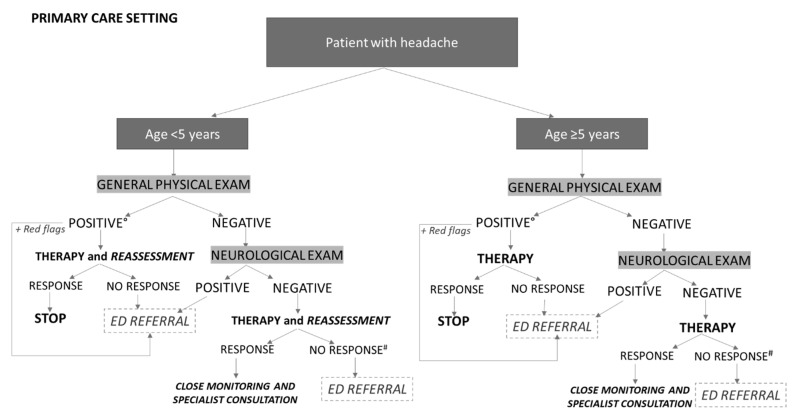
Management of pediatric headache in a primary care setting. ° General physical exam positive for non-life-threatening secondary headache that does not require further investigation. ^#^ Excludes somatoform disorders.

In the context presented in Answer C of Scenario 4, with a patient with headache and a general physical examination suggestive of secondary non-life-threatening causes (e.g., upper respiratory tract or gastrointestinal infections), the PCP should treat the underlying causes and conduct an initial analgesic therapy approach with a short-term follow-up, especially in younger children; in cases of worsening symptoms, therapy failure, or the appearance of warning signs or symptoms and/or positive neurological examination, ED referral is indicated (Figure 1) [4,28]. Similarly, a patient with a history of primary headache should be addressed to the ED in the case of recent onset of severe intensity headache, chronic persistent progression, or unresponsiveness to analgesics [23,28].

Therefore, it is proper to send the patient to the ED in the cases of Answers A, D, E, and G, as they present red flags, positive neurological objective examination, or failure to respond to first-line oral therapy.

**Recommendation** **3.** *The role of the PCP is to detect potentially dangerous or life-threatening conditions that require an immediate medical evaluation in an emergency setting. In the case of neurological alterations at physical examination, or in the presence of other warning signs, the patient should be immediately addressed to the ED. In the case of a general physical examination suggestive of secondary and non-life-threatening conditions, the underlying causes should be treated and an initial analgesic therapy approach should be performed under close monitoring, especially in preschool patients. ED referral is recommended only in the case of general worsening of symptoms, therapy failure, appearance of warning signs/symptoms, or positive neurological examination*.

##### First and Second Survey Results Comparison

In Scenario 3, answers D (dysautonomic signs preceding headache) and E (headache with temporal localization) remained scored without agreement, although the percentage of appropriateness for ED referral increased (from 36.1 to 45.8% and from 30.6 to 62.5%, respectively). On the other hand, the second survey results brought agreement for Answer H (headache with chronic progressive course), which was considered appropriate for ED referral by 75% of the participants.

In Scenario 4, the rate of appropriateness for answers A and G increased after the second survey (from 72.2% to 87.5% and from 63.9% to 87.5%, respectively). By contrast, the rating for inappropriateness for Answer B decreased (from 88.9% to 62.5%), with an increase in the number of uncertain responses. Strong disagreement remained for answers F and H, with percentages of appropriateness at 50% and inappropriateness at 37.5%.

#### 3.1.4. Recommendation 4 (Scenarios 5 and 6)

In the case of an acute headache recurrent for three months without red flags, the PCP should:

- Suggest a headache diary and refer the patient to a pediatric neurologist outpatient clinic;
- Suggest a headache diary and continue the follow-up, after OF evaluation;
- Prescribe an MRI with gadolinium;
- Prescribe a consultation to the pediatric neurologist;
- Prescribe a general ophthalmological evaluation; and
- Prescribe a cardiologist evaluation.

In the case of repeated episodes in an adolescent with acute moderate-intensity headache characterized by pulsating frontal pain and preceded by visual blurring, the clinic’s PCP should:

- Suggest ED referral; and
- Prescribe analgesic therapy and a pediatric neurology consultation.

As shown in the flow-chart in Figure 1, in the case of a child suffering with headache with normal general and neurological examination and no red flags, the PCP can suggest an antalgic therapeutic approach in the acute phase.

In younger children, it is appropriate to monitor the patient for at least an hour, or re-evaluate them after a few hours. In the case of improvement, it is indicated to plan a non-urgent pediatric neurologist consultation, maintaining close home monitoring with the family to detect any recurrence (more than four episodes/month) or worsening, that requires the specialist to move the consultation forward within 30 days. On the contrary, ED referral is recommended if symptoms worsen during observation. For patients older than 5 years, in the case of negative general and neurological examination, it is appropriate to recommend acute therapy for headache and collect a headache diary, with referral to the pediatric neurologist in the case of recurrence of episodes or therapy ineffectiveness. A headache diary helps the monitoring of the frequency of episodes and the temporal pattern, but also in identifying possible trigger factors [29]. A recurrence of at least five episodes per month is an indication for the PCP to request a deferrable urgent specialist referral.

In the case of failure of therapy in patients with negative general and neurological examination, it is important to also include in differential diagnosis psychosomatic/functional disorders, in which headache is a significantly frequent symptom in childhood [30,31]. The diagnosis of psychosomatic disorders is of exclusion. It can be suspected especially when, during the family and patient interview, relevant environmental and psychic elements appear, such as a history of physical or psychic trauma, psychic vulnerability, ongoing stressful conditions, and psychological or psychiatric comorbidities. A history of typical symptoms of psychosomatic disorders, such as abdominal, muscle and precordial pain, asthenia, or other neurological symptoms such as non-organic visual impairment, attention or memory difficulties, and dizziness may also suggest a psychosomatic disorder [31,32]. Of note, psychosomatic headache typically disappears with distraction. Psychosomatic headache should be distinguished from true somatic symptom disorders, in which one or more chronic somatic symptoms, not justified by any organic cause, cause disproportionate ideation and worry [33].

It is therefore fundamental for the PCP to inspect environmental and family dynamics, school performance, and the presence of psychosocial stressors, as they can be triggering factors of headache in primary or secondary to psychiatric/psychosomatic pathology. Furthermore, screening for alcohol, drug, smoking, smartphone, or video game abuse should be performed in all adolescent patients [34,35].

Regarding Answer B, OF cannot be considered the only discriminating factor that causes the PCP to refer the patient to the ED or a pediatric neurologist [21]. Similarly, the ophthalmologic consultation is not part of the routine management of recurrent benign headaches, except for cases of accompanying visual disturbances or suspected refractive or motility disorders. Anamnestic elements indicative of visual fatigue and refractive deficit can appear during the evening and after school, as well as ocular dryness.

With reference to Answer C of Scenario 5, the decision to perform neuroimaging should not be left to the PCP, but suggested by a pediatric neurologist consultant.

A cardiologist consultation (Answer F) does not represent a routine investigation in the outpatient management of benign forms of headache. It may be of diagnostic usefulness in differential diagnosis between the syncope and migraine with aura [36,37]. Sometimes, the neurologist could suggest performing echocardiography to search for patent foramen ovale. In other cases, transcranial doppler echocardiography may be requested. This exam allows to identify stenosis or occlusion of cerebral arteries, the passage of microemboli in cerebral circulation of at-risk patients (e.g., mechanical cardiac valve wearers), or to exclude blood flow through an interatrial septum defect as an underlying cause of a stroke or migraine with aura [38].

**Recommendation** **4.** *In the case of normal general and neurological examination, without red flags, after investigating the patient’s environmental context and presence of trigger factors, the PCP may recommend acute antalgic therapy, the use of a headache diary, and plan a clinical re-evaluation to assess symptom evolution and responses to therapy. Referral to a pediatric neurologist is recommended in the following cases: children under 5 years of age, lack of or insufficient response to therapy, worsening of symptoms, or in general, for a specialist diagnostic definition*.

##### First and Second Survey Results Comparison

With regard to Scenario 5, Response A obtained the agreement of panelists (with appropriateness increasing from 63.9% to 83.3%). The possibility of suggesting a headache diary collection after OF (response B) maintained an insufficient level of appropriateness and agreement (from 52.8% to 58.3%) with a corresponding slight increase in the percentage of inappropriateness. The indication to send the patient to the pediatric neurology expert obtained a higher percentage of appropriateness (from 44.4% to 66.7%), still not sufficient for agreement. Strong disagreement remained for Response E (ophthalmological consultation), with a reduction in the proportion of participants who considered this response appropriate (from 58.3% to 37.5%). Cardiologic consultation was also rated with disagreement (inappropriateness from 58.3% to 62.5%).

For Scenario 6, agreement was reached for the appropriateness of performing antalgic therapy and addressing the patient to a pediatric neurologist consultation (with an increase from 58.3% to 95.8%), while sending the patient to the ED was considered inappropriate by 62.5% of participants after the second survey (disagreement).

### 3.2. Emergency Department Assessment

#### 3.2.1. Recommendation 5 (Scenarios 1, 2, 3 and 4)

In the case of an adolescent with acute headache of moderate intensity, characterized by pulsating frontal pain and preceded by visual blurring, in ED it is indicated to request and prescribe:

- Analgesic therapy and discharge in the case of reduction of symptoms
- An urgent pediatric neurologist consultation
- An urgent CT scan in the first instance
- Ocular fundus
- Urgent ophthalmologist examination
- Hospitalization for clinical observation and MRI examination

In the case of headache characterized by pulsating occipital pain in adolescents, the following is indicated in the ED:

- Administration of analgesic therapy and discharge in the case of reduction of symptoms
- An urgent pediatric neurologist consultation
- An yourgent CT scan in the first instance
- Ophthalmologist consultation in the first instance
- Hospitalization for clinical observation and MRI examination

In the case of a first episode of high-intensity pulsating headache in an 11-year-old patient with difficulties in performing normal activities, without neurological deficits, and in the absence of a history of primary headache, the following is indicated in the ED:

- Analgesic therapy and discharge in the case of reduction of symptoms
- An urgent pediatric neurologist consultation
- An urgent CT scan in the first instance
- Ophthalmologist examination in the first instance
- Admission for clinical observation and MRI examination

In the case of a first episode of acute pressure-like frontal headache, in a 9-year-old patient, otherwise healthy, with an absence of fever, the following is indicated:

- Pain medication and clinical observation
- Performing OF
- Pain medication and discharge
- Pediatric neurologist elective consultation
- Pediatric neurologist urgent consultation

According to a recent systematic review, headache in children accessing EDs is primarily due to benign conditions that tend to be self-limiting or that can be resolved with appropriate pharmacological treatment; a wide variety of benign secondary etiologies are included, such as infectious events and post-traumatic headaches, which together with primary headaches, account for more than 60% of ED access [35,39]. Only 0.4–4% of pediatric patients present with forms of headache underlying intracranial pathologies associated with high morbidity and mortality [40,41,42].

After an adequate triage of the patient, according to the new five emergency codes indicated by the Italian Ministry of Health, the ED physician should look for anamnestic and objective red flags with the aim of performing appropriate urgent investigations for each condition and offering the right symptomatic therapy for primary headache forms. In ED, the assessment of OF should be included in a complete neurological examination, referring doubtful cases, when cycloplegia is necessary, to the ophthalmologist. The possible presence of intracellular and extracellular deposits, often calcified, at the level of the optic nerve papilla (drusen) often mimics optic nerve edema on OF examination [43,44], leading to the performance of unnecessary and invasive diagnostic tests. Drusen has an incidence of 0.4% in pediatric age and usually does not give clinical manifestations; however, visual field defects have been described among its complications [45]. In-depth tests, such as ultrasonography, fluorescein angiography and optical coherence tomography may be useful in making a correct diagnosis [46].

On the other hand, false negative findings on OF are possible in pediatric patients with headache [47,48]. For instance, in 48% of idiopathic intracranial hypertension (IIH) cases, one of the most important clinical signs, papilledema, was absent [49]. Sixth cranial nerve palsy has been reported to be associated with IIH in 46–60% of cases, and loss of visual acuity in 6–20% of cases [50,51]. In pre-school children, in whom primary forms of headache are less frequent and examination of the OF, as well as complete neurological objective examination are difficult to perform, observation and clinical monitoring play an important role [13]. Finally, the coexistence of visual symptoms not clearly correlated with migraine aura is another indication for performing ophthalmologist consultations [21].

If ICHD-3 criteria for primary headache disorder are met, no further investigations are necessary. In the presence of one or more high-risk factors, however, performing neuroimaging is appropriate, whereas if relative risk factors are present, it may be appropriate to take a more restrained approach according to the individual context. Some neurological manifestations, such as visual disturbances (reduced vision, visual field narrowing, scintillating scotomas), sensory or speech disturbances, and focal paresthesias may be related to an aura if developed within several minutes, and last less than an hour [21,25].

In the case of a negative physical exam and lack of response to therapy, in the suspicion of headache secondary to psychosomatic disorder, in an ED regimen, it is appropriate to undertake a brief observation to assess the progress of symptoms, generally self-limiting, independently from administered therapy. The general management depends on the significance of the overall picture, in terms of intensity of symptoms and impact on quality of life, and it is generally referred to the pediatrician. If the pervasiveness of the disorder is limited and the symptoms are mainly reactive, an outpatient management is possible, while in the case of chronic symptoms, with a likely psychopathological component, it is essential to start psychological and neuropsychiatric support, in agreement with the family that will take part in it.

Among the investigations to perform in the diagnostic evaluation of acute headache, blood tests are not particularly useful, except for defining possible infectious causes or underlying hematological alterations [52].

Lumbar puncture is not routinely recommended, but should be performed in children with suspected infectious, hemorrhagic, or expansive intracranial processes, after adequate evaluation of clinical contraindications (vertebral abnormalities, ongoing seizures, cardiorespiratory instability, suspected infection at the sampling site, severe thrombocytopenia with ongoing bleeding, suspected meningococcal sepsis). If a central nervous system infection is suspected, lumbar puncture gains priority over neuroimaging to avoid therapeutic delays [53].

In an emergency setting, electroencephalography (EEG) in a pediatric patient with headache has little usefulness; therefore, it should be limited in the differential diagnosis with epilepsy, based on pediatric neurologist advice [54].

The most useful investigation to differentiate secondary headache forms remains to be neuroimaging, which, in ED, should be reserved for patients with high-risk red flags. In fact, in patients with a normal neurological examination, significant pathological findings were found in only 1–2% of cases [55,56,57,58].

Consultation with the pediatric neurologist in the ED, where possible, is a useful form of support if red flags are present at the first evaluation. In the absence of red flags, where the history depicts a primary form of headache, the ED physician may recommend an elective specialist evaluation.

Scenario 1 presents an adolescent with headache that, in terms of intensity, location, and associated symptoms, does not raise obvious suspicions of secondary forms for which urgent ophthalmologist consultation or neuroimaging is required. The ED pediatrician may evaluate the OF as an integral part of the neurological physical examination and observe the response to symptomatic therapy, choosing for admission and/or elective neuroimaging if there is no response, or if significant signs or symptoms appear.

In Scenario 2, in the absence of more detailed data, it is appropriate to request a pediatric neurologist consultation and to perform an accurate physical examination which, if positive, will lead to the indication for admission and execution of nuclear magnetic resonance imaging (MRI).

With reference to Scenario 3, the presence of the first episode with high intensity, abnormal localization, and difficulties in performing normal activities should alert the ED pediatrician. Indeed, in adolescents, forms of secondary headache may present with isolated symptoms, like learning and attentional problems, changes in personality, lethargy, apathy, or symptoms of depression [18]. It is possible to attempt pharmacological therapy and observe the clinical response, but in the case of persistence or worsening of these symptoms, a pediatric neurologist consultation is recommended.

In Scenario 4, if no other warning signs are present, the described manifestations may point to a primary headache. In fact, tensive forms of headache are most often expressed in children as bilateral, severe pain. It is therefore advisable to prescribe analgesic therapy and discharge the patient recommending an elective specialist evaluation.

**Recommendation** **5.** *During triage, patients not requiring urgent care should be distinguished from those with red flags suggestive of life-threatening disease. In ED, laboratory tests are indicated in the case of a positive general physical examination for secondary forms or when a benign form (e.g., infectious) is suspected, in order to define etiological therapy. Ophthalmologic consultation should be considered whenever a visual symptom is identified and is not clearly correlated with primary headache, or in all cases that require an ocular fundus in cycloplegia. Urgent consultation with a pediatric neurologist is indicated, whenever possible, in cases presenting neurological signs or symptoms; it may be deferred to an elective consultation in cases of headache symptoms suggestive of primary or secondary non-life-threatening forms that can be treated with symptomatic therapy.*

#### 3.2.2. First and Second Survey Results Comparison

With reference to Scenario 1, only answers A and C obtained significant agreement among panelists (appropriate for 83.3% and inappropriate for 87.5% of participants, respectively). In responses B, E, and F, participants who rated these options inappropriate prevailed, even if with insufficient rates for agreement (54.2% to 62.5%). The option of performing an OF in this scenario was considered appropriate by 66.7% of participants, with an increase of 22.3 percentage points.

In Scenario 2, concerning headache of occipital localization, Answer B was considered appropriate by 75% of participants, and C inappropriate by 75% of participants. The remaining responses regarding discharge after a symptomatic therapy response, request for ophthalmologist consultation, and hospitalization with performance of MRI did not obtain the agreement of the participants, especially for Response E, with considerable uncertainty (33.3%),

Scenario 3 obtained agreement only for Response C regarding the inappropriateness of an urgent CT. There was an increase in the number of participants who considered answers A and B (58.3% and 66.7%, respectively) appropriate, while considerable discordant opinions were derived from responses D and E, related to the need for an eye examination and hospitalization for MRI.

In Scenario 4, after the second survey, agreement on the appropriateness of response A regarding pain treatment followed by clinical observation was consolidated, with values close to agreement (70.8% of appropriateness) as compared to the possibility of discharging the patient after therapy response (66.7% appropriateness). The percentage of participants who considered the appropriateness of performing an OF increased (from 52.8% to 66.7%). The indication of sending the patient to an elective neurologist consultation obtained a level of appropriateness very close to agreement (70.8%).

#### 3.2.3. Recommendation 6 (Scenarios 5, 6, 7 and 8)

In the case of unilateral, high-intensity pulsating headache in a 5-year-old patient with difficulty in performing normal activities, without neurological deficits and in the absence of a history of primary headache, in emergency settings, the following is indicated:

- Admission and observation
- Admission and observation in the case of fever or systemic symptoms
- Discharge with referral to pediatric neurologist clinic
- Discharge if symptoms subside during observation, with referral to pediatric neurology expert in the case of recurrence

In the case of moderate-intensity headache, hospitalization is required if:

- Pre-school age child
- Chronic-progressive course of headache
- Acute recurrent course of headache
- Association of weight loss
- Association of weight gain
- Familiarity with ischemic events during youth
- Association with upper respiratory tract infection/gastroenteritis
- Association with vomiting on awakening

Hospitalization is indicated in patients with headache and:

- Negative first level imaging and persistence of pain
- Negative first level imaging and indication for further diagnostic investigation in urgency
- Indication for brain MRI in election for further investigation
- Patient diagnosed with migraine and with attack lasting for more than 72 h responding to iv therapy administered in the ED
- Seven-year-old patient with reported bilateral non-pulsating headache with negative neurological examination but pain intensity 6–7/10 VAS, partially responsive to analgesic therapy
- Seven-year-old patient with bilateral mild-to-moderate, non-pulsating headache and history of minor frontal head trauma 4 days before, in the absence of other neurological signs

Hospitalization is indicated in the cases of:

- Headache in patient >5 years of age with negative neurological examination and failure to respond to acute therapy
- Headache in patient <5 years of age with negative neurological examination and failure to respond to acute drug therapy
- Headache at any age with positive neurological examination (focal signs, nystagmus, decreased visual acuity, lethargy, or irritability)
- Headache with positive physical examination for acute infectious disease
- Therapy-responsive headache associated with fever and upper airway infection, in patient >5 years of age
- Recent-onset headache, defined as being mild and responsive to therapy, in patients >5 years of age
- Recent-onset headache of high intensity
- Headache with persistent vomiting, even in the morning
- Headache with nocturnal awakenings

The main guidelines on the management of acute headache in the literature date back about 10 years [58,59,60]. None of these guidelines clearly defines the hospitalization criteria of a pediatric patient with headache. A systematic review carried out by the Pediatric Headache Commission of the Italian Society of Pediatric Neurology (SINP) showed that the available guidelines are of poor quality and not homogeneous: some of them focus on an emergency setting, while others focus on an outpatient setting [60]. Currently, it is possible to define recommendations only on the basis of clinical experience and low levels of evidence.

**Figure 2:**
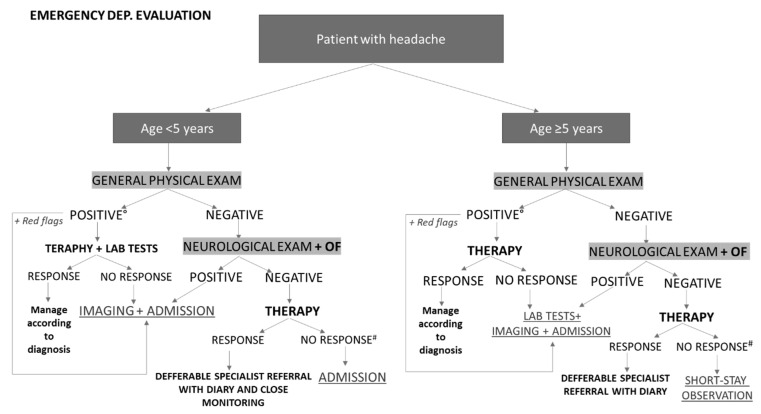
Management of pediatric headache in emergency department and hospitalization criteria. ° General physical exam positive for non-life-threatening secondary headache that does not require further investigation. ^#^ Excludes somatoform disorders.

Thus, the expert panel defined the following approach for ED pediatricians asked to evaluate a child with acute headache (Figure 2). In preschool-aged patients, in the case of a positive general physical examination for secondary forms of headache, without signs or symptoms for life-threatening forms, it is advisable to perform analgesic therapy and first-level examinations (e.g., blood tests in the case of signs of infection), and to evaluate whether to perform further investigations based on the specific diagnostic suspicion, or to discharge the patient with specific therapy in the case of headache remission and a negative familiar/personal history for possible secondary causes. It is suggested to maintain close monitoring at the primary care pediatrician, in order to refer to a child’s neurology specialist cases of recurrence or worsening of symptoms. The presence of a positive neurological examination and/or positive OF for papilledema and/or other red flags indicate the need for urgent neuroimaging and hospitalization. In the absence of all these signs, only acute treatment of headache is indicated, with hospitalization in the case of persistence/worsening of symptoms, rather than a referral to a pediatric neurologist in the following 2–4 weeks in the case of remission [5,61]. In the case of non-responsiveness to therapy, headache secondary to psychosomatic disorders should always be included in differential diagnosis.

In children over 5 years of age, the recommended approach is similar, preferring a short-stay observation in the case of a failure of analgesic therapy, with negative general and neurological objective examination and negative OF. In the case of a positive general objective examination without life-threatening signs or symptoms, it is indicated to postpone further diagnostic investigations, depending on severity and individual cases.

According to what has been said, cases similar to Scenario 5 are therefore worthy of hospitalization, even in the absence of fever and systemic symptoms. In Scenario 6, there are significant red flags for hospitalization in answers A, B, D, F, H. With reference to Scenario 7, cases in answers A, B and C deserve hospitalization. Similarly, the cases proposed in answers B, C, D, G, H, I of Scenario 8 require hospitalization to perform in-depth investigations.

**Recommendation** **6.** *Hospitalization is indicated for patients who, irrespective of age, present with a positive general physical examination for benign secondary forms whose causes require further diagnostic and therapeutic investigation and/or alterations on neurological examination and/or fundus oculi indicative of papilledema and/or red flags. In preschool patients (<5 years old), it may be appropriate to hospitalize even forms with negative neurological and general examination but an absent response to symptomatic therapy. A short observation regimen may be planned for similar cases in school-aged patients (>5 years).*

##### First and Second Survey Results Comparison

In reference to Scenario 5, characterized by a 5-year-old patient with a first episode of unilateral headache of high intensity without other red flags, participants reached agreement for the appropriateness of admission for observation in the case of fever or other systemic symptoms (79.2%). The option of discharging the patient with advice to receive a pediatric neurology consultation, if there was a reduction in symptoms during observation, was rated appropriate for 62.5% of members of the second survey.

In Scenario 6 (hospitalization in the case of moderate-intensity headache), there was an increase in the percentage of appropriateness for responses A and B and inappropriateness for response C. Disagreement remained for the response related to weight gain (50% appropriateness vs. 33.3% inappropriateness). Finally, agreement was obtained for answers B and F, with appropriateness of 79.2% and 83.3%.

Even in Scenario 7, the level of agreement is adequate only for Response B in the analysis of the results of the second survey. Responses that do not reach agreement refer to hospitalization with elective brain MRI performance, a partial response to pain-relieving therapy in headache without red flags, and mild to moderate post-traumatic headache.

Among the answers in Scenario 8, only the one related to hospitalization in the case of non-response to acute pain therapy in patients without red flags and headache associated with acute infectious disease failed to reach agreement. In Round 2, Response G also achieved agreement for appropriateness of hospitalization (79.2%).

#### 3.2.4. Recommendation 7 (Scenarios 9, 10 and 11)

In a 10-year-old child with morning headache preceded by vomiting and nausea, without focal signs or nuchal rigidity, with negative ocular fundus, the following is indicated:

- Performance of an urgent brain CT scan
- Admission and MRI examination
- Pediatric neurology expert assessment
- Clinical observation for 12–24 h
- Execution of blood tests to assess nutritional status and infections
- BMI calculation
- Blood pressure and ECG detection

In the case of elevated blood pressure associated with headache and/or irritability depending on age:

- Perform urgent brain CT scan
- Perform deferred urgent MRI of the brain
- Perform cardiologic evaluation and echocardiogram
- Pediatric neurology expert assessment
- Perform OF and ophthalmologist consultation

In the case of paroxysmal rapid progressive headache (pulsating, diffuse throughout the skull) partially responsive to NSAIDs, associated with visual disturbances and vomiting preceded by nausea and, on physical examination, nuchal rigidity one should:

- Perform blood tests, including coagulation
- Perform lumbar puncture
- Perform fundus oculi
- Perform brain CT scan
- If family history of migraine is present, consider deferred neuroimaging

According to some studies reported in the literature, the risk of an expansive brain lesion increases in the case of a recent headache onset (especially if less than 6 months), nocturnal episodes or onset on awakening, association with vomiting, altered neurological examination, an altered state of consciousness, and the absence of a family history of migraine [12,38].

A systematic review suggests that urgent neuroimaging should be performed only when high-risk red flags are present, whereas a more restrained approach, appropriate to the single case, is suggested in the case of low-risk red flags or an absence of red flags [40]. A study conducted on neuroimaging studies performed in an emergency setting in the presence of red flags with normal neurological examination showed that the prevalence of urgent intracranial abnormalities is small, around 1%, due to the low specificity of these signs; on the contrary, the probability of a positive neuroimaging result is higher in the presence of red flags and altered neurological examination (ataxia, focal seizures, cranial nerve dysfunction, nystagmus, abnormal reflexes) [56]. The conclusion of the expert panel is that in the case of a positive general physical examination for secondary headache, the presence of a positive neurological examination and/or an OF indicative of papilledema and/or other red flags give an indication of urgent neuroimaging and hospitalization.

A distinct diagnostic pathway should be defined in suspected stroke, whose clinical presentation is age- and site-dependent. In infants and young children, it may be nonspecific, with few focal clinical signs and symptoms more frequently represented by seizures, irritability or headache. In infancy and later ages, the predominant clinical presentation consists of an acute neurological deficit associated or not with seizures. The rapidity of symptom onset strongly correlates with the pathogenesis of the ischemic event [62]. If an ischemic stroke is suspected, a brain CT scan or, if available, an MRI should be performed within one hour of the child’s arrival in the ED (gold standard) [63]. The goal is to distinguish an ischemic lesion from a hemorrhagic one as soon as possible. The CT is the most widely used investigation because of its short execution time and greater availability in urgent situations. The MRI is the gold standard for both initial lesion detection and follow-up; it should be performed in association with angiography-MRI within 24 h of admission if the initial CT scan is negative but stroke is still suspected [64,65].

Another exception is an alleged CNS infection, for which it is usually indicated to proceed directly with spinal tap before urgent imaging, to avoid delays in antibiotic therapy. In the case of signs of intracranial hypertension (such as papilledema or focal neurological signs), a new-onset seizure, an immunodepressed state, altered consciousness, coagulation disorders, skin infection at the site of inoculum, or an encephalic CT scan should be performed first [66,67].

In Scenario 9, morning-type headache associated with nausea and vomiting should not always be considered a high-risk form: in fact, these signs may be present in forms of primary headache [21]. Given the school age (>5 year) and negative neurological findings, the recommended approach is to administer analgesic therapy and observe the response, performing first-line tests if necessary. In the case of non-response, persistence, and/or worsening of symptoms, the performance of neuroimaging, in this case, MRI, is inappropriate.

In Scenario 10, acute headache and/or irritability in a patient with high systolic/diastolic blood pressure for age, sex, and height may indicate a hypertensive crisis or baseline hypertension, especially if previous headache episodes are documented in the patient’s history. On a general physical exam, vascular hypertension is frequently found in patients with intracranial hypertension, cerebral edema, or cerebral herniation. In an emergency setting, therefore, urgent encephalic CT is indicated [55]. A cardiac evaluation and echocardiogram should be prescribed in the case of negativity of the above-mentioned tests to exclude cardiac causes underlying the arterial hypertension, which in turn could explain the headache [4].

The headache episode in Scenario 11 is paroxysmal and progressive (pulsating, diffuse throughout the skull) and should be an alarm bell for the clinician because it is potentially indicative of meningitis or intracranial hemorrhage. If the systemic picture is not suggestive of infection, the partial responsiveness to medical therapy and the association with visual disturbances, vomiting, and nuchal rigidity are additional warning symptoms for which it should be indicated to perform an urgent CT brain scan or MRI where available, as well as blood tests, including coagulation screening [18]. In these cases, lumbar puncture can be considered a second-level examination if, after a negative CT scan for expansive, traumatic, or vascular processes, the physical examination remains persistently altered, in order to exclude infectious/inflammatory processes or idiopathic intracranial hypertension, as illustrated in Figure 1.

In conclusion, in emergency settings, CT is the imaging of choice because it is more accessible and does not require sedation compared to the MRI [68]. Moreover, the CT is useful in clinically unstable patients with specific implanted devices for whom MRI is contraindicated. Specific scanning protocols are used to reduce the radiological risk of CT, and radiant dose parameters can be optimized according to patient size and age [69,70].

**Recommendation** **7.** *CT scan without contrast medium is the test of choice in an emergency setting, due to its rapidity of execution and its easier accessibility in the ED, without the need for patient sedation. In the case of suspected stroke, the brain MRI should be performed within one hour of patient arrival in the ED*.

##### First and Second Survey Results Comparison

Agreement was obtained for responses D, E, and G of Scenario 9 (75%, 75%, and 79.2% of appropriateness, respectively); in contrast, responses A, B, and F did not reach agreement, although, particularly for response A, the percentage of inappropriateness increased from 41.7% to 62.5%.

In Scenario 10, participants considered the performance of an urgent CT scan of the brain (79.2%) to be appropriate compared with a deferred urgent MRI (50%) in cases of hypertension with headache and/or irritability. Below the agreement limits for appropriateness (70.8%) was response D, related to the involvement of the child’s neurology specialist.

In Scenario 11, there was a slight decrease in the appropriateness of lumbar puncture; however, for above 70%, agreement was obtained for the inappropriateness of response E.

### 3.3. Hospital Admission

#### 3.3.1. Recommendation 8 (Scenarios 1 and 2)

In a 10-year-old child with morning headache preceded by vomiting and nausea, without focal signs or nuchal rigidity, the following is indicated:

- CT execution
- RMN execution
- Evaluation by the pediatric neurology expert
- 12–24 h of clinical observation
- Performing blood chemistry tests to assess nutritional status and ongoing infections
- BMI calculation
- BP and ECG detection

In cases of headache with acute confusional state in children or adolescents associated with agitation and aphasia, one should:

- Exclude encephalitis in the first instance by radiologic investigation
- Perform lumbar puncture
- Conduct child neuropsychiatric consultation to rule out psychoaffective/somatization disorders
- Perform viral serological investigations
- Observation to assess possible resolution within 24 h before continuing with the diagnostic procedure
- Electroencephalogram

When a child with headache is admitted to the ward, it is crucial to re-evaluate the patient periodically and to continue the diagnostic process to differentiate life-threatening secondary headache forms from “benign” secondary headache forms and primary headache forms. As already mentioned, the majority of cases of secondary headache requiring hospitalization in pediatric age is related to systemic infectious/inflammatory or post-traumatic processes, and quite rarely related to expansive processes. In the case of Scenario 1, the presence of vomiting and nausea preceding the attack in a school-aged child with no clear neurological signs could place a primary form in differential diagnosis, such as basilar migraines, with an acute gastroenteritis or an expansive process. Clinical observation for at least 24 h, together with vital parameter monitoring allows to appreciate the evolution and to direct laboratory and instrumental tests. In suspected intracranial hypertension, pressure monitoring is useful during the hospital stay to assess for hypertension [18]. A basilar migraine is defined by the coexistence of migraines with neurological symptoms originating from the brainstem, or simultaneously from both cerebral hemispheres. Diagnostic criteria include dizziness, visual disturbances in both hemispheres, aphasia and dysarthria, sensory alterations, and ataxia. Symptoms of aura may develop gradually or occur in succession for more than 5 min (5–120 min) and with rapid resolution. Therefore, basilar migraines should be placed in differential diagnosis with stroke and posterior cranial fossa pathologies [71,72]. A prolonged confusional state may also occur in non-convulsive status epilepticus, which may present motor manifestations limited to automatisms. In these cases, the diagnosis is confirmed by EEG examination [73].

In contrast, BMI calculation is not diriment to the diagnostic process, but remains a useful parameter in cases with a history of malnutrition, eating disorders, or excess weight. The preferred neuroimaging examination is the MRI, as discussed in recommendations 7 and 10 [67]. The headache episode in Scenario 2 describes a new-onset acute headache that therefore requires a careful search for possible red flags. In consideration of a confusional state and focal neurological signs such as aphasia, we should first exclude an inflammatory, vascular-based, or intracranial expansive process by performing blood tests and urgent neuroimaging (CT scan or MRI if available) and, if CNS infection is suspected, these tests should be preceded with a spinal tap, if not contraindicated. It will, in addition, be appropriate to perform serologic investigations based on clinical suspicion [11,18]. The use of EEG is not routinely recommended, but is useful to differentiate epileptic-like manifestations [53]. If the criteria for primary headache (e.g., basilar migraine) are not met, it is useful to investigate possible psychoaffective/somatization disorders by child neuropsychiatric examination only in the case of exclusion of life-treating infectious or expansive processes.

**Recommendation** **8.** *During hospitalization, close monitoring of parameters and clinical conditions is appropriate to assess the patient’s response to therapy and possible appearance of “red flags” for secondary headaches. The choice of diagnostic investigations depends on clinical suspicion and is supported by the evaluation of the pediatric neurologist. In the case of a positive general examination, it is recommended to perform microbiological and specific instrumental examinations to exclude infections. The appearance of neurological warning signs during hospitalization is an indication to perform urgent neuroimaging to exclude life-threatening conditions*.

##### First and Second Survey Results Comparison

Round 2 resulted in agreement for almost all responses in Scenario 1, except for Response B (performance of MRI).

There was a lack of agreement for Scenario 2. However, appropriateness prevails with regard to the indication for lumbar puncture (70.8%), child neuropsychiatric examination to distinguish somatoform disorders (66.7%), and EEG (66.7%).

#### 3.3.2. Recommendation 9 (Scenarios 3 and 4)

In the case of metamorphopsia, as well as micro- and macropsia, and spatio-temporal distortions preceding the headache, the following are useful:

- Perform urgent brain CT scan
- Perform deferred brain MRI
- Perform cerebrospinal fluid examination to exclude infectious processes
- Perform EEG to rule out occipital epilepsy
- If not associated with other symptoms, do not perform further investigations
- Ophthalmologist consultation

Epilepsy should be placed in differential diagnosis with primary headache/migraine:

- When symptoms begin rapidly
- When there is mild confusion/sensory numbness that resolves after headache
- In the case of nausea and vomiting
- In the case of white/black, zigzag and/or central aura
- In the case of aura lasting <5 min
- In the case of sudden-onset colored circular aura

The differential diagnosis between some epilepsy syndromes and primary headache may require special attention. For instance, occipital epilepsies (such as Gastaut’s epilepsy, with onset between 3 and 15 years) are characterized by visual hallucinations (usually colored elementary figures, mostly circular, with rapid vertical movements) of short duration followed by a post-critical headache. These visual symptoms mimic a migraine aura that instead manifests with a black-and-white fortification spectrum, with a zigzag appearance, or scotoma, with slower progression and a latero-lateral direction [74]. Other visual manifestations of migraine aura are less common in pediatric age, including metamorphopsias (distorted vision) and micro/macropsias (abnormally small or magnified vision of objects). A comprehensive ophthalmologist consultation should be asked in these cases to rule out retinal problems. Autonomic symptoms, such as nausea and vomiting, are recurrent in pediatric headaches, but are also characteristic of focal epilepsy, such as Panayiotopoulos epilepsy, an idiopathic form that usually begins between 3 and 6 years of age [75].

Therefore, it will be appropriate to perform a pediatric neurologist consultation and an EEG. Neuroimaging has low sensitivity, being both migraine and idiopathic epilepsies of benign nature.

**Recommendation** **9.** *In the differential diagnosis between primary headache and epilepsy, it is useful to consider some characteristic clinical features and to consult a pediatric neurologist who may request an EEG.*

##### First and Second Survey Results Comparison

In Scenario 3, with the exception of the appropriateness of performing an EEG (100%) and a general ophthalmologist examination (75%), agreement was not reached for the remaining responses.

Uncertainty and disagreement also prevailed in Scenario 4, which dealt with the differential diagnosis between epilepsy and primary headache, without significant differences between the first and second rounds except for answers A and F, where the percentage of appropriateness increased considerably.

#### 3.3.3. Recommendation 10 (Scenario 5)

MRA (MRI with angiography) should be requested:

- If posterior reversible encephalopathy (PRES) is suspected
- In the case of family history of coagulopathies or vascular malformations
- In the case of suspected idiopathic intracranial hypertension
- In the case of suspected familial hemiplegic migraine
- Routinely in the presence of any red flag
- In the case of post-traumatic headache

According to the main international guidelines, inpatient neuroimaging should be considered in the presence of red flags suggestive of etiology secondary to life-threatening diseases: positive family history for cerebrovascular disease, coagulopathies, malformations or genetic neurological disorders, detection of neurological warning signs or symptoms (especially in the case of focal signs or alteration of alertness or consciousness), recent onset, unilateral pulsating headache of severe intensity (thunderclap), chronic progressive evolution, or different and more intense manifestations than the episodes reported in the patient’s history [55,76,77].

The American Board of Internal Medicine and American Headache Society (AHS) claims that MRI, when available, is preferable to CT in the evaluation of headaches not in an emergency setting, especially in suspected lesions of sella, craniocervical junction, posterior fossa, white matter abnormalities, congenital malformations, and infectious processes [78]. In fact, this examination, although more expensive and longer in terms of image acquisition time, minimizes radiation exposure and is more sensitive than CT in these cases. However, in young children, MRI may require sedation [68]. Please refer to the 2018 Expert Panel on Neurological Imaging guidelines for more details [68].

MRI with angiography is recommended in cases of suspected headache secondary to life-threatening disease, particularly if vascular (ischemic- or malformative-based). The use of gadolinium as a contrast agent has been widespread for years and commonly applied to pediatric age. Some studies have shown that gadolinium administered in pediatric age is able to deposit in some organs, such as encephalon and bone, but the clinical implication has not yet been determined [79,80,81,82]. Therefore, the advice of pediatric radiology experts is to use the contrast agent in moderation.

When evaluating imaging to be performed when vascular abnormality is suspected, it is also suggested that both angio-CT and MRA examinations be considered if subarachnoid or parenchymal blood is identified in a previous urgent CT or MRI [68].

Items appearing in responses A, B, C, D, F describe items worthy of in-depth neuroradiological diagnostic investigation.

**Recommendation** **10.** *Red flags identified through the collection of anamnestic data, and general and neurological physical examination should direct to in-depth examinations through neuroimaging. In an inpatient setting, an MRI is preferred in the suspicion of lesions of the saddle, craniocervical junction, posterior fossa, white matter abnormalities, congenital malformations and infectious processes, as it is more sensitive than CT and safer in terms of radiation protection. For the evaluation of ischemic events or vascular malformations, it is recommended to consider CT angiography or MRA in addition to the standard sequences depending on availability and level of urgency.*

##### First and Second Survey Results Comparison

In Scenario 2, responses E and F, relating to the indication for MRA in the presence of any red flag (with an increase in the percentage of appropriateness from 36.3% to 50%) or in the case of post-traumatic headache, are devoid of agreement, and opinions were distributed rather homogeneously.

### 3.4. Acute Treatment

#### 3.4.1. Recommendation 11 (Scenario 1)

In the case of acute migraine in a pediatric patient without clear diagnosis, as a first-line therapy it is possible to give:

- Paracetamol or ibuprofen
- Paracetamol, given its more rapid action as compared to ibuprofen
- Ibuprofen, given its more rapid action as compared to paracetamol
- Ibuprofen and triptans (almotriptan, eletriptan, rizatriptan, sumatriptan, zolmitriptan) in patients younger than 12 years old

The target of the treatment of acute migraine in childhood should be an adequate response, with minimum side effects and a return to usual activities as fast as possible [83]. According to the last AHS guidelines, the acute treatment of migraine is based on oral analgesics as needed, given the rapidity of action of this route of administration [28]. In the guidelines, there is a strong recommendation regarding the efficacy and safety of non-steroidal anti-inflammatory drugs (NSAIDs) in children. Ibuprofen at a dose of 7.5–10 mg/kg, is particularly comparable to 10 mg/kg of paracetamol in reducing headache, but shows better rapidity of action, with moderate levels of evidence regarding the efficacy in resolving the episode by two hours, especially when administered orally [28,84,85]. A Cochrane of 2016, analyzing some randomized controlled trials on a pediatric population of 7630 children, showed that ibuprofen is better than a placebo in reducing headache, but there was no statistically significant difference regarding the risk of recurrence and the resolution of the associated neurovegetative symptoms [86]. The data regarding the efficacy of paracetamol in resolving the episode in two hours are contrasting [28]; however, a recent trial by Pavithra et al. on a population of 50 children did not show a statistically significant difference in terms of safety and efficacy between ibuprofen at the dose of 10 mg/kg and paracetamol at 15 mg/kg [87]. In clinical practice, the two drugs are equally used for acute migraine, depending on the subjective response of the patient.

A third line is represented by naproxen at a dosage of 5 mg/kg, which is used much less for patients of pediatric age.

Acute treatment should be administered early, and it is possible to administer it a second time if migraine shows up again in 24 h to treat recurrences. The preferred route of administration in the absence of side factors (like vomit and dehydration) is the buccal one, thanks to his great efficacy, rapidity of action, and easy management. It may be necessary to try more than one drug before finding the most effective one for every single patient: if the first drug may result in lower efficacy, one should try a second one of the same pharmacological family before passing to a different one (this is more valuable for triptans, discussed below). Education of families as to the right use of the best analgesic to treat every single episode is indicated; it means that, for a single patient, it may be necessary to use different drugs for attacks with different characteristics [86,88].

**Recommendation** **11.** *In the management of acute headache attack in pediatric patients with no definite diagnosis, ibuprofen is a first-line therapy for his rapidity of action. As an alternative, it is possible to use paracetamol, the efficacy of which is similar to that of ibuprofen, despite lower rapidity. The preferred route of administration is the oral one, using buccal formulations.*

##### First and Second Survey Results Comparison

In this scenario, a higher percentage of participants that rated Answer B as inappropriate and Answer C as appropriate was registered (from 72.2% to 83.3% and from 72.2% to 87.5%, respectively), gaining agreement for both of the answers.

#### 3.4.2. Recommendation 12 (Scenario 2)

In the case of acute pain in a patient with a clear diagnosis of migraine, it should be explained to the patient that to treat associated symptoms (nausea, vomiting, phono- and photophobia):

- The most commons symptoms (such as phonophobia, photophobia, nausea and vomiting) regress with analgesics
- The oral method of administration should be the favorite one for better efficacy
- In the case of phonophobia and photophobia, there is a satisfying response to ibuprofen, particularly if taken at the beginning of symptom onset
- In the case of nausea and vomiting, an antiemetic treatment should be associated
- In the case of nausea and vomiting, triptans grant a good response
- The treatment of nausea and vomiting should be avoided since they regress spontaneously and in a short time

Associated symptoms, such as phono- and photophobia, nausea, and vomiting are frequent during an acute migraine attack or an episode of chronic migraine exacerbation [1]. After excluding these symptoms as referable to red flags, the physician should explain to the patient that they regress with analgesics, and it is usually more effective if taken at the beginning of symptoms or when they are of low to moderate intensity [88]. Ibuprofen shows the best response as the first line treatment (low level of evidence in AHS guidelines). In clinical practice, paracetamol is also used, although its real effectiveness in reducing associated symptoms is debated [28]. For migrainous forms, many studies have shown the efficacy of triptans versus placebo in treating associated symptoms besides the migraine itself, with a satisfying response in 2 h [28]. It may be possible and advisable to associate antiemetic treatment in order to promote oral analgesic therapy for those whom vomiting makes that route of administration difficult. In the case of persistence of emetic symptoms, it is important to refer the patient to an ED in order to gain venous access, start intravenous rehydration, and administer antiemetic and analgesic therapy.

**Recommendation** **12.** *In the case of acute attack in a patient with diagnosis of migraine, in order to treat associated symptoms (phonophobia, photophobia, nausea and vomiting), physicians should explain to the patient that they regress with analgesics taken at the beginning, or when pain is still low to moderate. The oral way of administration is the advisable one in buccal formulations. It is possible to associate an antiemetic treatment in order to promote oral therapy; if nausea and vomiting make the oral route difficult, it may be necessary to refer the patient to the nearest ED to gain venous access and administer fluids, as well as analgesic and antiemetic therapy.*

##### First and Second Survey Results Comparison

After the second round, all answers gained agreement for their accuracy, except for Answer F, regarding the possibility of discouraging the treatment of nausea and vomiting (inadequate for 54.2% of members).

#### 3.4.3. Recommendation 13 (Scenario 3)

With reference to the triptans approved in pediatric age:

- All of the drugs of this class, approved in pediatrics, either oral or nasal, are equally effective
- Drugs given via nasal route should be preferred because they have shown a better relation between benefits and adverse effects in comparison to those given orally
- In the case of phono- or photophobia, there is no relevant improvement of symptoms
- They are used in combination with NSAID drugs
- They are used in combination with NSAID drugs in the case of an insufficient response to a single triptan
- In Italy, children older than 10 years are approved
- In Italy, children older than 12 years are approved
- It is possible to use the sumatriptan nasal spray, zolmitriptan nasal spray, and rizatriptan as tablets
- It is not possible to use them off-label in children younger than 12, even if under a specialist prescription

There are four triptans commonly used in pediatric age: rizatriptan, almotriptan, sumatriptan, and zolmitriptan (Table 7). Their use is approved for children older than 12 years, and one, rizatriptan, for children older than 6 years. For those patients with a confirmed diagnosis of primary headache, not fitting the approved classes of age, the off-label prescription of a triptan is possible on the advice of a pediatric neurologist.

Their use is generally reserved to acute migraine attacks not properly responding to NSAID drugs and/or paracetamol. They should be taken when pain is still mild to moderate with a rapid and satisfying response, even though they may be effective in moderate to severe pain [26,89,90,91,92]. Triptans are available in two formulations: oral (almotriptan, rizatriptan, sumatriptan/naproxen) and nasal spray (zolmitriptan), both showing the same efficacy, even if the oral one is better tolerated [86].

The AHS systematic review showed that the strongest scientific evidence about headache resolution at one and two hours was for nasal zolmitriptan. Nasal sumatriptan also seems effective, but only at a dose of 20 mg [28]. Moreover, zolmitriptan and sumatriptan/sodium naproxen seem effective at treating associated symptoms, such as phonophobia or photophobia, with a clear improvement at two hours from administration [86]. Unfortunately, their overall frequent use in chronic forms can reduce the time it takes for it to be effective on a migraine based on drug abuse; in these cases, it is possible to use a drug containing sumatriptan and sodium naproxen in order to improve efficacy and to reduce the risk of a drug abuse-induced migraine. A systematic review by Oksui et al. showed moderate to high evidence of the efficacy of combined therapy, even with low doses of Sumatriptan [28]. Adverse events include asthenia, dizziness, vertigo, dry mouth, lethargy, flushing, and heat sensation, and they are generally rare. They cannot be prescribed in the case of ischemic cardiovascular pathologies and disorders of cardiac conduction with the presence of an accessory method. In the case of an insufficient response to a monotherapy with a triptan, guidelines suggest the association with a NSAID drug, particularly ibuprofen (Table 8).

**Recommendation** **13.** *Triptans are indicated in adolescents with migraine, possibly in association to ibuprofen in the case of insufficient response to monotherapy. There are four triptans approved in pediatric age: almotriptan, sumatriptan, and zolmitriptan for children older than 12 years, and rizatriptan for children older than 6 years. Off-label use for younger ages is possible under pediatric neurologist prescription. Their use is restricted to acute pain not responding sufficiently to treatment with non-steroidal anti-inflammatory drugs and/or paracetamol, and they should be taken when pain is still low to moderate for a rapid and safe response, even though they may be still effective in cases of moderate to severe pain. They are available in oral and nasal formulations. Both methods of administration showed similar efficacy, even though the oral one is better tolerated.*

##### First and Second Survey Results Comparison

The lack of agreement between answers A and B, referring to the efficacy of triptans based on the route of administration, and Answer D (use of Triptans combined with non-steroidal anti-inflammatory drugs). Among the other responses that acquired agreement, it is noteworthy that the one about the impossibility of off-label use in children younger than 12 was considered inadequate for 79.2% of participants after the second survey.

#### 3.4.4. Recommendation 14 (Scenario 4)

Among antiemetic drugs for treatment of associated symptoms:

- The use of ondansetron in pediatric age is indicated
- The use of dopamine agonists in pediatric age is indicated
- Their use is indicated in the case of acute headache of unknown diagnostic definition

During acute migraine attacks or in the case of exacerbation of chronic headache-associated symptoms, such as nausea and/or vomiting are commonly seen, and they make oral analgesic therapy difficult, increasing the patient’s discomfort. It is demonstrated that these symptoms regress with analgesic therapy alone and that the efficacy is stronger and the effect more rapid when it is administered at the beginning of symptoms or when the pain is still of low to moderate intensity [12]. Whenever therapy with NSAIDs or triptans is not able to fix these symptoms or vomiting does not allow for adequate oral treatment, the most recent guidelines promote the use of antiemetic drugs in order to improve symptoms and to promote oral therapy administration, even in the case of headaches of unknown origin (Table 9) [93].

In pediatric age, the ondansetron, serotonin receptor antagonist, and dopamine agonists like promethazine and proclorperazine are approved for use; the last ones are also considerable as an alternative therapy for acute migraine but with severe adverse effects, such as extrapyramidal symptoms [18,94]. Ondansetron is widely used in pediatric clinical practice and in oncohemathology overall, and some evidence exists about their efficacy in cyclic vomit syndrome [95]. A retrospective study that compared ondansetron and dopamine agonists in an ED department showed similar efficacy for both [96]. In a retrospective study that compared the efficacy of promethazine, metoclopramide and proclorperazine for acute headache and associated symptoms, it was shown that the first one was more frequently associated to therapeutic failure [97]. The use of these drugs in Italy is mainly restricted to hospitals.

**Recommendation** **14.** *Even though analgesic treatment alone is shown to control associated symptoms, the administration of antiemetic drugs can be considered with a double effect of limiting nausea and vomiting, often associated with headache, and promote oral administration of analgesics when necessary. The main drugs approved in these conditions are ondansetron, serotonin receptor antagonists, used in hospitals, and dopamine antagonists, such as promethazine and proclorperazine.*

##### First and Second Survey Results Comparison

Disagreement on antiemetic drug administration was confirmed except for ondansetron, which retained adequacy for 91.7% of participants in the second round.

#### 3.4.5. Recommendation 15 (Scenario 5)

If an adolescent with a history of migraines comes to the ED for a new attack and they are not responding to NSAIDs or Triptans:

- Intravenous fluid hyperhydration should be started
- Metoclopramide can be efficient in treating both nausea and headache
- NSAID intravenous therapy should be attempted
- If the headache lasts more than 24 h, it is possible to use dihydroergotamine
- If the headache lasts more than 72 h, it is possible to use dihydroergotamine

In the case of patients with a history of migraines and coming to the ED for acute attacks, and they are not responding to NSAIDs and triptans, venous access should be established in order to start intravenous hyperhydration and to administer endovenous paracetamol or NSAIDs (ibuprofen or ketorolac) [26,97]. Metoclopramide, a dopamine antagonist, is a possible alternative to analgesics, that showed efficacy in treating both headache and associated gastrointestinal symptoms (dose: 0.2 mg/kg, maximum dose 10 mg) [98]. More specifically, metoclopramide can prevent dehydration and poor absorption of oral drugs by contrasting vomiting and gastric stasis that compromises analgesics absorption (Table 10). However, the use of metoclopramide in Italy is contraindicated in children younger than 16, after a multicentric study of the Health National Institute in 2004 showed there was a high risk of adverse neurological effects, particularly extrapyramidal signs [99].

In the case of acute migraines lasting more than 72 h (status migrainosus), administration of intravenous dihydroergotamine at a high (0.5–1 mg/kg every 8 h) or low (0.1–0.2 mg/kg every 8 h) dose is suggested [100,101,102,103]. Before administering this prolonged therapy, it is necessary to:

1. Perform a test dose (administer half dosage, adequate for weight and age);
2. Perform a pregnancy test, when necessary;
3. Treat with antiemetics 20 min before therapy (proclorperazine or metoclopramide for the first three doses, then, if still needed, it is suggested to use ondansetron, due to the high risk of extrapyramidal effects of dopamine antagonists).

**Recommendation** **15.** *In children presenting with acute migraine attack, not responding to first-line treatment, or status migrainosus, venous access should be established to start intravenous hyperhydration and intravenous paracetamol or NSAID (ibuprofen or ketorolac) therapy. Metoclopramide is a dopamine antagonist alternative to first-line analgesics, that showed efficacy in treating both vomiting and pain. In the case of status migrainosus, the therapy consists in intravenous administration of dihydroergotamine at high (0.5–1 mg/kg every 8 h) or low (0.1–0.2 mg/kg every 8 h) doses.*

##### First and Second Survey Results Comparison

In Scenario 5, agreement was reached for all answers except for A, indicating the use of intravenous hyperhydration was considered adequate for 66.7% versus 38.9% of participants in the first round.

### 3.5. Prophylaxis

#### 3.5.1. Recommendation 16 (Scenario 1)

Prophylactic treatment of migraine should be recommended when:

- Attacks are of high intensity with interference to quality of life, or they have a frequency of >4 days/month with 3–4 migraine attacks per month for at least 3 months
- Attacks respond only to acute polytherapy
- There is poor responsiveness to acute treatment with increased school absence or access to the ED
- In the case of medication-overuse headache

For headache prevention, most children benefit from behavioral and lifestyle modification. Prophylactic pharmacological or bio-behavioral treatment should be considered when headache occurs with sufficient frequency and severity, causing migraine-related disability [28,104]. According to the Italian guidelines drawn up in 2003 and approved by the Italian Society for the Study of Headache, the Italian Society of Childhood and Adolescent Neuropsychiatry, and the Italian Society of Pain Clinicians [24], the rationale for prophylactic drug therapy in pediatric headache is:

- Attacks of high intensity or that which follow one another with high frequency (more than four headache days per month and 3–4 migraine attacks per month for at least three months);
- Reduction of the patient’s quality of life;
- Poor responsiveness to acute treatment with disability (i.e., influence on school attendance and play/sport activities, frequent visits to ED) [28,105,106].

For the assessment of headache disability, the short questionnaire “Pediatric Migraine Disability Assessment” (PedMIDAS) can be used; this questionnaire measures the functional impact of headache over three months with just six questions [104]. Adolescents with migraine who have a PedMIDAS score above 30 (moderate to severe migraine with associated disability) have a higher risk of developing mood and anxiety disorders, with increased severity and frequency of migraine [24,28]. Alternatively, in adolescents, the Headache Under-Response to Treatment questionnaire (HURT) can be administered [107].

Few data exist on medication-overuse headaches in children [108], but this type of secondary headache is a real threat, especially for adolescents with migraines. Acute therapy overuse might make migraines less responsive over time and facilitate the development of chronic migraine.

**Recommendation** **16.** *The indications for preventive therapy in pediatric headache are: attacks of high intensity or those occurring with high frequency (more than 4 days/month with 3–4 headache attacks/month for at least three months), reduction in patient’s quality of life, and poor responsiveness to acute treatment with disability. The decision to undertake preventive therapy is a responsibility of the pediatric neurology specialist. Prophylaxis may also prevent adverse events of drug abuse, such as medication overuse headache.*

##### First and Second Survey Results Comparison

Most of the participants (75%) considered Response B (indication for prophylactic therapy when responding only to acute polytherapy) to be appropriate, in contrast to the indications of the main guidelines. Agreement was also obtained for Response D on medication-overuse headaches, with an increase in the percentage of appropriateness from 47.2 to 79.2%.

#### 3.5.2. Recommendation 17 (Scenario 2)

In the absence of major comorbidities, prophylactic drug therapy of migraine in a pediatric patient can be carried out with:

- Propranolol
- Amitriptyline alone
- Amitriptyline + cognitive-behavioral therapy
- Topiramate
- Valproate
- Triptans

The new guidelines issued by the AHS [29] include topiramate, propranolol, amitriptyline in combination with cognitive-behavioral therapy (CBT), and cinnarizine as drugs for the prophylactic treatment of migraines (Table 11).

Specifically:

- Among antiepileptics, topiramate at a dosage of 2–3 mg/Kg/day up to 100 mg/day was more effective than the placebo in reducing seizure frequency, and is the only drug indicated by the Food and Drug Administration for treatment of headache in adolescents aged 12–17 years [109,110]. However, it is no more effective than placebo in reducing headache attacks by at least 50% or in reducing disability [109,111]. Therefore, there is insufficient evidence for its efficacy in the prevention of pediatric headache. Its efficacy has been demonstrated in adults [112] but not in the pediatric population, for whom data in the literature are scarce. Nevertheless, topiramate may be indicated in male patients with certain forms of epilepsy associated with headache [113].
- Propranolol is a beta-blocker frequently used in pediatric populations, including for headache prevention, requiring adequate monitoring of blood pressure and heart rate [114,115]. Its dosage is 20–40 mg/day. The possible increase in blood concentrations of some triptans during prophylactic propranolol therapy is noteworthy [116]. In a systematic review, propranolol was found to have a better risk–benefit profile than topiramate, but there were no statistically significant differences in safety [117].
- Cinnarizine is an antihistamine and L-type calcium channel blocker. It is not recommended for co-administration with calcium antagonists in patients with current or previous depressive illness, arrhythmic heart disease, obesity, liver failure, pyramidal disorders and is contraindicated under 12 years of age. In the AHS guidelines, cinnarizine has been shown to be moderately effective compared to placebo in reducing the frequency and severity of migraine attacks, with low levels of evidence of efficacy in reducing migraine episodes by more than 50% compared to the placebo [28].
- Although some studies, such as the 2017 Childhood and Adolescent Migraine Prevention (CHAMP) Study [26,110], question the role of amitriptyline alone in headache prevention, the combination of amitriptyline (dosage: 1 mg/kg/day) and CBT has been shown to be effective in reducing the frequency of headache episodes and may be the first choice in patients whose headache causes a drastic reduction in quality of life or in adolescents with depression and anxiety disorders [118]. However, physicians should advise against the use of amitriptyline in patients with ongoing depression, as they are particularly at risk of developing suicidal ideation [28].
- The use of triptans remains limited to the management of acute attacks [18,28].

**Recommendation** **17.** *The current guidelines only provide the use of topiramate, propranolol, amitriptyline + CBT and cinnarizine for the prophylactic treatment of migraine, for which the analysis of the evidence has given satisfactory results in terms of efficacy.*

##### First and Second Survey Results Comparison

In Scenario 2, responses regarding the appropriateness of propranolol and amitriptyline prophylactic drug therapy in combination with CBT reached agreement (75% and 79.2%). Seventy-five percent of the experts considered the use of CBT alone inappropriate. Disagreement remained for the use of valproate, triptans, and topiramate (the latter was considered appropriate by 62.5% of participants).

#### 3.5.3. Recommendation 18 (Scenario 3)

In the case of pre-existing anorexia, the following drugs should be avoided as prophylactic therapy:

- Topiramate
- Valproate
- Amitriptyline
- Propranolol

The decision on which medication to use for preventive drug treatment is based on comorbidities, adverse drug effects, and parents’ and patient’s preferences [24]. Physicians should discuss the evidence of efficacy for topiramate for the prevention of migraines in children and adolescents and its side effects in this population with the patient and their family [24]. Adverse effects of topiramate include the following: increased incidence of anorexia, increased episodes of upper airway inflammation, weight loss, gastroenteritis, paraesthesia, drowsiness, difficulty concentrating, angle-closure glaucoma, and urinary stones. Because of these findings, topiramate should be used with caution when anorexia is suspected in the patient, or the diagnosis is already present [28]. Therefore, based on the patient’s comorbidities, topiramate is more frequently used for epileptic patients, due to its anticonvulsant effects, or obese patients, due to its anorectic effect. With reference to other correlations between the prophylactic drug and patient’s comorbidities: valproate is preferred in males with epilepsy, as it has anticonvulsant effects and teratogenic adverse effects (see below), propranolol is used with caution in asthmatic patients due to its non-selective beta-blocking mechanism, and in diabetic or depressed patients, amitriptyline is used mainly in patients with anxiety disorders or depression [24,28].

**Recommendation** **18.** *To date, efficacy data of the main drugs used for headache prophylaxis are controversial. The choice of the appropriate drug is based on comorbidities, adverse effects, and parents’ and patient’s preferences.*

##### First and Second Survey Results Comparison

Contraindication to the use of topiramate in the case of pre-existing anorexia is considered appropriate by 83.3% of participants. In contrast, the drugs considered in the other answers did not reach agreement.

#### 3.5.4. Recommendation 19 (Scenario 4)

If a migraine prophylactic therapy is agreed with the patient and family:

- Start on low doses, gradually increasing the dosage over 3–4 weeks, even in the case of side effects if therapeutic benefit is achieved
- Start with low doses, gradually increasing the dosage over 3–4 weeks, discontinuing therapy in the case of side effects even if a therapeutic benefit is achieved
  Therapy should be continued for 12 weeks if a benefit is achieved
- Therapy should be continued for 6–12 months if there is a benefit.

There is little data on when to stop preventive treatment, and risk of relapse after discontinuation is variable. Preventive drug therapy should be started at the lowest possible dose. Doses should then be gradually increased over three to four weeks until a therapeutic benefit develops, the maximum administered dose is reached, or significant side effects occur [24]. Treatment should be continued for seven to eight weeks at the correct effective dose to achieve a real benefit. If migraine episodes decrease in frequency and intensity, therapy should be continued for approximately 6 to 12 months [24,107,117,119]. If preventive drug therapy fails, it is possible to change the class of drug used, or to recommend neurostimulation or CBT in combination with amitriptyline. The physician should inform patients and their parents about the risks and benefits of discontinuing preventive drug therapy once clinical improvement has been achieved [28,117].

**Recommendation** **19.** *Preventive drug therapy should be started at the lowest possible dose. Doses should then be gradually increased over 3–4 weeks until a therapeutic benefit develops, the maximum administered dose is reached, or significant side effects occur. Treatment should be continued for 7–8 weeks at the correct effective dose to achieve a real benefit. If migraine episodes decrease in frequency and intensity, therapy should be continued for approximately 6–12 months.*

##### First and Second Survey Results Comparison

There is considerable disagreement in Scenario 4 regarding the method of titration and assessment of the adverse effects of prophylactic drugs. With regard to therapy duration, agreement prevailed on continuation for at least 6–12 months (83.3% appropriateness).

#### 3.5.5. Recommendation 20 (Scenario 5)

In adolescents going to a primary care pediatrician clinic and reporting occasional, severe headaches without vomiting/nausea or other warning signs, the following actions are indicated:

- A headache invalidity assessment should be carried out
- Take a careful personal and medication history
- Suggest the compilation of a headache diary
- Seek advice from the pediatric neurologist
- Recommend the use of opioids when pain is not controlled with NSAIDs/paracetamol
- Recommend use of nutraceuticals as needed
- Set up a prophylaxis with nutraceuticals lasting 2–3 months

In the management of recurrent headache without other associated symptoms and warning signs, as presented in recommendations 1–4, the PCP should carefully collect the patient’s history, monitor the evolution by suggesting a headache diary, and use the PedMIDAS headache questionnaire to define the disabling effects of headache on the patient’s life. The use of opioids is discouraged because of the risk of reinforcing the pathogenetic mechanism of headaches, making it chronic and encouraging the abuse of these medications, especially in at-risk populations, such as adolescents [18,28,80]. Specialist advice from a pediatric neurology expert can be given after adequate follow-up if therapy is insufficient or ineffective and if there are doubts about the pattern of presentation, or simply for a more accurate diagnostic definition.

The use of nutraceuticals has become widespread in clinical practice, including non-specialist settings. Although there is no clear evidence of their efficacy in children and they are not mentioned in the most recent guidelines, nutraceuticals are often used in the prevention of headache, particularly in mild and occasional forms, when parents prefer not to start other therapies. Among these, riboflavin, magnesium and melatonin are the most widely used, since they are considered safe and well-tolerated (Table 12) [107,120]. Riboflavin (vitamin b12) is a cofactor in oxidation-reduction reactions in the electron transport chain within the mitochondria, which some theorists believe is involved in the pathophysiology of migraine [121]. Riboflavin is the most extensively studied supplement for pediatric patients, but the evidence for its ability to reduce the frequency and intensity of migraine attacks is not statistically significant. Low levels of evidence have been demonstrated in adulthood [122]. Despite this, many pediatric neurologists prefer to use it because of its good tolerability. Recommended doses range from 25 to 400 mg/day [122].

The correlation between low magnesium levels and tension-type and migraine headaches has been proven for several years. Among the pathogenic hypotheses, magnesium deficiency could have an effect on excitatory synapses, and thus on cortical spreading depression, or promote the intracellular pro-inflammatory cascade and vasoconstriction [123]. Despite inconsistent results on its efficacy in preventing headaches [124], its use, especially in the form of magnesium pidolate at dosages of 1.5–4.5 g, has not been shown to be related to significant adverse reactions and is considered by many experts to be a valid supportive therapy, especially in females of pubertal age [125].

Melatonin is known for its chrono-biotic, anti-inflammatory, and antioxidant properties. Mainly used in sleep disorders and favored by the virtual absence of side effects, it has also been used in prevention and acute treatment of headaches, but clinical data are not significant [125,126].

In the management of associated symptoms, in particular nausea and vomiting, supplements of natural origin, especially ginger-based, have been increasingly used in recent years, but the evidence for their efficacy is still weak [127].

**Recommendation** **20.** *The use of nutraceuticals in the preventive treatment of headache remains debated. In clinical practice, nutraceuticals are not contraindicated in mild primary headaches or in support of drug therapy, considering their good tolerability and safety.*

##### First and Second Survey Results Comparison

With regard to the use of nutraceuticals, the expert panel did not reach agreement on the use of nutraceuticals in acute therapy or as prophylaxis in primary headache cases referred to the PCP. In such cases, there was also disagreement about the need to refer the patient to a pediatric neurologist (54.2% appropriateness).

#### 3.5.6. Recommendation 21 (Scenario 6)

In headache prevention, hygiene measures include:

- Sleeping for a few hours in the afternoon
- Instructing children to go to bed and wake up at around the same time
- Regular mealtimes are recommended (usually three meals/day)
- It is recommended that children have a snack before going to bed
- Reduction of physical activity and sports is suggested

Clinicians should explain to patients and families that lifestyle and certain behavioral factors may influence the frequency of headache, and they should educate patients and families to identify and modify factors that act as triggers for headache (overweight, alcohol and caffeine use, lack of physical activity, insufficient hours of sleep, exposure to tobacco smoke) [28,107]. Hygiene measures to be taken in the case of chronic childhood headache are:

- Sleep hygiene: it is advisable to sleep an adequate number of hours per night and to maintain a routine sleep–wake rhythm. Insomnia, snoring, and frequent night waking can worsen the frequency of attacks.
- Physical activity: regular exercise in children has not been shown to be effective in preventing migraine, although studies in adult patients show the reverse. Physical activity of at least 30 min 3–5 times a week is recommended.
- Adequate water intake: increasing daily fluid intake has been shown to reduce the frequency and intensity of migraine attacks. Fluid intake varies according to age and level of physical activity, but includes 8–10 cups of non-caffeinated drinks per day.
- Regular food intake: fasting or skipping meals may act as a trigger for migraine. Children should have three meals/day.
- Identification of triggers: triggers should be identified as early as possible and avoided. Migraine occurs most often during school time, so the child’s school history should always be assessed in cases of frequent migraine in children. Caffeine and alcohol are known triggers for migraine and should be avoided. Some premonitory symptoms of migraine (fatigability, changes in mood, neck stiffness and increased sense of hunger) can often be misinterpreted as triggers [26,128].

**Recommendation** **21.** *Physicians should explain to patients and families that lifestyle and certain behavioral factors can influence the frequency of headache and should educate patients and their families to identify and change factors that act as triggers for headache.*

##### First and Second Survey Results Comparison

After the second round, all answers in this scenario reached agreement.

#### 3.5.7. Recommendation 22 (Scenarios 7 and 8)

For an adolescent girl undergoing prophylactic drug therapy for migraine with aura, the pediatrician should:

- Recommend biofeedback
- Discontinue valproate or replace it with another prophylactic drug
- Recommend CBT
- Discourage competitive sporting activity
- Recommend reducing alcohol and caffeine intake
- Do not recommend oral contraception

In the case of an adolescent patient of childbearing age who has been prescribed valproate as a preventive therapy for migraine, the pediatrician should:

- Discuss with the girl and her parents the teratogenicity of valproate, thus explaining the risk of possible pregnancy
- Change the drug used as preventive therapy
- Prescribe a gynecological examination and transvaginal ultrasound every six months
- Require periodic monitoring of blood valproic acid levels

As mentioned in Recommendation 21, overweight, alcohol and caffeine use, and lack of physical activity are some of the conditions that may worsen headache [26,28,107,128]. Competitive sports activity is not contraindicated.

The combined use of complementary therapies is often a successful strategy, as demonstrated by the effectiveness of the combination of amitriptyline and CCT [119]. Among the forms of complementary therapies, the most internationally accepted are:

- Behavioral-based therapy: according to Italian guidelines, this type of treatment can be considered as a first-choice approach or as a supplement to symptomatic and prophylactic drug treatment in pediatric patients, especially in the case of parental and/or young patient resistance to the use of drugs, presence of psychiatric comorbidity (anxiety disorders, mood disorders, social phobia, sleep disorders, etc.), presence of family issues, and ineffectiveness or inadequate response to previous treatments [57].
- Cognitive-behavioral therapy: CBT aims at teaching patients behavioral strategies to cope with pain, prevent migraine episodes through regular use of relaxation exercises, and assist patients with migraine-associated disabilities and comorbidities such as anxiety and depression. Its efficacy has been demonstrated especially in combination with amitriptyline in reducing headache-related disability, and the frequency of attacks by at least 50 percent of actual headache days [119]. According to the 2003 Italian guidelines, this type of treatment can be considered as a first choice in pediatric patients in whom headache causes significant disability or decreases their quality of life [24]. However, CBT alone may also be considered an effective first-choice therapy in certain patients, on pediatric neurologist advice [28]. Often, biofeedback (BFB) is included into CBT during the teaching of relaxation exercises, thus allowing patients to see the physiological changes that occur after relaxation exercises.
- BFB: This is a technique through which a subject receives information and can about its biological state and can change it. BFB thus creates an external link through which the subject can keep one or more physiological functions under control. BFB uses an electronic instrument to monitor these functions and sends information to the patient about its progress in the form of an acoustic or light signal. In this way, the patient can become aware of the progresses on these functions, which are usually considered independent of willpower, and can therefore learn to control it. According to some studays, patients undergoing BFB have experienced an improvement in migraine frequency and a decrease in attack duration and intensity [129]. Although this technique appears to be effective, it is not widely used due to several limitations: high cost, low availability, few dedicated staff, and low compliance of young patients.

For the management of adolescent girls on valproate therapy, see Recommendation 23.

**Recommendation** **22.** *The use of complementary therapies for headache prevention is a possible alternative to pharmacological prophylaxis. In certain patients, on the advice of the specialist, it may be considered an effective first-line therapy. Complementary therapies may also be useful in situations of poor patient and parental compliance, poor tolerance and/or presence of contraindications to drug therapy.*

##### First and Second Survey Results Comparison

Responses on the use of BFB, valproate withdrawal and contraindication to oral contraception did not receive agreement, although the first two were considered appropriate by 54.2% and 62.5%, and the last was inappropriate for 62.5% of participants. In Scenario 8, answers regarding therapeutic switch from valproate to a different drug in childbearing age patients and periodic monitoring of valproate blood level did not reach agreement despite high values of appropriateness (70.8% for both).

### 3.6. Follow-Up

#### 3.6.1. Recommendation 23 (Scenarios 1 and 2)

During the follow-up of a female adolescent suffering from migraines, the PCP should:

- Tell the patient to inform them any time a new migraine-related symptom appears
- Refer the patient to a child neurologist if she is taking topiramate or valproate as a prophylactic treatment to evaluate changes in therapy or to assess the teratogenic risk
- Recommend a gynecological evaluation if the patient wishes to start contraceptive oral therapy
- Periodically evaluate the patient’s health, especially if the migraine has a negative impact on mental health

A patient on topiramate as a prophylactic drug for migraines. may be referred to a neurologist in the case of:

- Persistence of headache symptoms during the first month of treatment
- Onset of anorexia
- Asthma exacerbation

Prophylactic treatment for migraines must be continued at the right dosage for seven to eight weeks to achieve a beneficial effect, regardless of the drug. It is necessary to wait for two months before suspecting a failure of response and refer the patient to a specialist for reassessment [28,107]. Otherwise, the PCP must pay attention to significant adverse reactions, inadequate tolerance to the drug, or rapid changes in the headache pattern during the first few months.

Regarding the drug of choice, the physician must consider the teratogenic effects of valproate and topiramate when prescribing a prophylactic treatment to girls of childbearing age: valproate should be avoided, as the European Medicine Agency advises to use Valproate only as a last choice, and adverse effects of topiramate must be disclosed to female patients before starting therapy [28,130,131].

The PCP must adequately inform the patient of contraceptive options, remembering that valproate (at a dosage >200 mg/day) could potentially reduce the efficacy of hormonal oral therapy; gynecological evaluation and folic acid integration are recommended [131,132].

Increase in upper airway infections and anorexia are some of the adverse effects of topiramate, the first being a possible cause of asthma exacerbation. If the above-mentioned effects appear, the patient should be referred to a pediatric neurologist. Other adverse effects are weight loss, paraesthesia, sleepiness, trouble with concentration, closed-angle glaucoma, gastroenteritis, and kidney stones.

**Recommendation** **23.** *A specialist re-assessment should be recommended every time a patient taking prophylactic therapy shows significant adverse reactions, inadequate tolerance to the drug, or rapid changes in headache patterns during the first few months.*

##### First and Second Survey Results Comparison

Regarding the question in Scenario 1, in contrast to previous results, the majority of the experts (87.5%) stated as appropriate the necessity to refer the adolescent to a child neurologist in therapy with valproate or topiramate for a therapeutic switch.

Scenario 2 only showed agreement on Answer B (presentation of anorexia during prophylactic therapy with topiramate) with 83.3% of appropriateness.

#### 3.6.2. Recommendation 24 (Scenario 3)

Home blood-pressure monitoring:

- Is indicated in every patient suffering from headache
- Is indicated in the case of metabolic syndrome
- Is indicated in the case of high BMI
- Is indicated when headache is associated with neurovegetative symptoms
- Must be associated with heart-rate monitoring
- Is mandatory in children <6 years old with headache
- Is indicated in patients taking propranolol
- Is indicated in patients on propranolol, if presenting with syncope
- Is indicated in patients taking amitriptyline

The first steps in the evaluation of a patients suffering from headache is accurate physical examination, and blood pressure and HR measurement: these can rule out headache secondary to hypertension or hypertensive crisis [11,13]. If primary or secondary hypertension is suspected, home blood pressure monitoring is recommended.

Among the health conditions presented in the scenario, neurovegetative symptoms and age <6 years are not criteria-indicative for blood-pressure monitoring (even if a child cardiologist may recommend it in some cases).

Hypotension and bradycardia are among the adverse effects of beta-blockers; these drugs are preferred in patients suffering from hypertension and tachycardia [133]. In this case, HR and orthostatic blood pressure must be monitored, and it should normally be above 60 bpm after one minute of physical exercise. Slow deescalating dosages and blood pressure monitoring are indicated before discontinuing therapy to avoid hypertension.

Calcium channel blockers and amitriptyline can cause hypotension too; blood pressure monitoring is mandatory for these drugs [57,134].

**Recommendation** **24.** *Physical examination of a patient with headache must include heart rate and blood pressure assessment. Evidence from the literature do not give precise indications on blood pressure monitoring in patients with headache but it is considered mandatory in patients assuming beta blockers, calcium channel blockers and amitriptyline.*

##### First and Second Survey Results Comparison

Agreement on home blood pressure monitoring has been reached on the answers about metabolic syndrome, high BMI, and association with HR measurement in patients taking beta-blockers and amitriptyline (with percentages ranging from 75% to 95,8%). No agreement was reached on the option of monitoring it in all patients suffering with headache, presenting neurovegetative symptoms or for patients <6 years of age.

#### 3.6.3. Recommendation 25 (Scenario 4)

In order to prevent headache from medication overuse and refer the patients to a pediatric neurologist re-evaluation, acute treatment should be taken:

- No more than 14 times/month for oral analgesics
- No more than 2 times/week for oral analgesics
- No more than 8–9 times/month for triptans
- No more than once a week for triptans

Acute treatment of headache pain overuse has been linked to medication overuse headache in adult patients [135]. Adults suffering from migraine with more than six attacks/month have a higher risk of chronic headache, and thus tend to overuse symptomatic therapy.

Medication overuse headache is a concrete risk in the pediatric age (especially in adolescents), even if there is not great evidence in the literature. A similar link has been hypothesized between the migraine attack frequency, excessive use of analgesics, and progression towards chronic headache. This can be avoided with patient and family education [136].

The time limits for medications use are:

- Analgesics: no more than 14 times/month;
- Triptans, ergotamine, opioids: no more than nine times/month (opioids are cited with regard to ICHD-3, but their use is not recommended);
- Combinations of drugs: for less than a three-month period.

If the frequency of the attacks is high or the response is insufficient, the patient must be referred to a pediatric neurologist for preventive therapy prescription.

**Recommendation** **25.** *Medication overuse headache is a concrete risk in pediatric age. The PCP must monitor acute treatment in patients with headache. Assumption of NSAIDs for more than 14 days/month, triptans/ergotamine for more than 9 days/month, combined drugs for more than 3 months are criteria for a specialist re-evaluation and start of a preventive therapy.*

##### First and Second Survey Results Comparison

In relation to medication overuse headache, Answer C got 95.8% of appropriateness. Answers B and D did not reach agreement but showed an increase in inappropriateness rate (58.3% and 70.8%, respectively).

## 4. Discussion

A summary of the developed recommendations is reported in Table 13.

The clinical management of headache in children and adolescents has long been underestimated, firstly by international societies, whose guidelines and recommendations gave less relevance to pediatric age [3,37,107]. However, the rise of pediatric ED admissions for headache and its associations with multiple comorbidities require an appropriate organization and adequate healthcare professionals training with the aim of developing an efficient network between primary care pediatricians, hospital pediatricians and pediatric neurologists (Table 14).

The RAND method allowed involving a group of professionals among PCPs and hospital pediatricians with expertise and awareness of the local impact of the above-mentioned issues, so to define the appropriateness of procedures with low level of evidence.

In view of the variety of headache presentation as well as the number of risk factors for secondary headache, 39 scenarios were developed to encompass as many conditions as possible. The scenarios were grouped in the six steps of the diagnostic-therapeutic process: primary care evaluation, ED evaluation, hospital admission, acute therapy, prophylaxis and follow-up.

### 4.1. Primary Care Pediatrician Evaluation

From data analysis of primary care evaluation phase, a good knowledge of red flags among participants was found from the first round of the questionnaire, while disagreement persisted on the referral to ED for patients with dysautonomic symptoms or partial response to acute therapy.

In fact, most of the answers of Scenario 1 and 2 reached the agreement from the first round. Noteworthy, the appropriateness of macrocephaly as red flag raised from 61.1% to 91.7% after meeting clarification of the at-risk age range.

In Scenario 3, no agreement was reached for the referral to ED in the case of dysautonomic signs preceding headache or temporal localization of headache, despite an increase in appropriateness (from 36.1% to 45.8% and from 30.6 to 62.5%, respectively). Agreement was found on the referral to ED in chronic progressive headache (75% of final appropriateness).

The indication for an urgent evaluation in ED revealed disagreement also in Scenario 4, presenting a patient with dysautonomic signs and partial response to monotherapy (62.5% of inappropriateness). Lack of agreement resulted also for answer F and H, concerning an episode of likely migraine with aura and an episode of migraine not responding to paracetamol, respectively.

The recommendation on headache diary in Scenario 5, after performing an OF, obtained an insufficient level of agreement (from 52.8% to 58.3% of appropriateness). A higher rate of appropriateness was reached by the indication of a pediatric neurologist referral (from 44.4% to 66.7%), albeit not sufficient to determine agreement. Similarly, disagreement resulted for the inappropriateness of a cardiologist consultation (from 58.3% to 62.5%). Moreover, the indication to a complete ophthalmic examination found strong disagreement, with a decrease in appropriateness rate from 58.3% to 37.5%.

With reference to the clinical case of Scenario 6, that describes a likely primary headache, the indication to acute therapy and subsequent referral to a pediatric neurologist was rated as appropriate with agreement (95.8%), while the indication to ED referral was rated inappropriate for the 62.5% of participants.

A careful application of ICHD-III criteria could help in these cases of discordance due to misinterpretation of signs and symptoms of migraine [3,36,109].

### 4.2. Emergency Department Evaluation

In relation to the management in ED, the survey results showed a lack of agreement for the indications to OF, ophthalmologist consultation, urgent imaging in the case of morning headache with dysautonomic symptoms or hypertension, as well as hospitalization criteria relative to acute therapy response.

In Scenario 1, a case of headache preceded by blurred vision and without red flags, the answers that obtained disagreement concerned the indication to a neurologist and ophthalmologist consultation, as well as the admission to perform an MRI (inappropriateness ranging from 54.2% to 62.5%). The indication to an OF was rated appropriate for the 66.7% of panelists, with a rise of 22.3% after the meeting.

In Scenario 2, describing an occipital headache, the indications concerning the discharge after acute therapy effectiveness, the ophthalmologist consultation and hospitalization for neuroimaging study did not reached agreement. On the other hand, participants came to agreement for appropriateness of a pediatric neurologist consultation and inappropriateness of an urgent CT.

With reference to Scenario 3 (high intensity headache with impairment of daily life activities), only the inappropriateness of an urgent CT obtained agreement, while remarkable disagreement resulted from the requirement for an ophthalmologist consultation or admission for second level imaging. Of note, there was an increase in participants who rated appropriate the discharge of drug-responsive patients and the referral to a pediatric neurologist (58.3% and 66.7% respectively).

In Scenario 4, after second round, the appropriateness of acute therapy followed by clinical observation reached the 70.8% of appropriateness as compared to the 66.7% for the option of discharge in the case of headache resolution. Lack of agreement remained for the indications to an OF and a non-urgent neurologic consultation (the latter receiving a 70.8% rate of appropriateness).

With reference to the appropriateness of hospitalization criteria, in scenarios 5, 6, 7 and 8 the panelists come to agreement for: a preschool child with fever and systemic symptoms with unilateral high-intensity headache (79.2%), recent onset high-intensity headache (79.2%), chronic-progressive headache (79.2%), family history of ischemic events in youth (83.3%), positive neurological examination (100%), non-response to acute therapy (95.8%), negative first line imaging but need for urgent second level exams (87.5%), association to early-morning vomiting (91.7%), nocturnal awakenings (83.3%). weith loss (79.2%).

Disagreement resulted for the indications of discharge with non-urgent neurologist referral in the case of preschool child responsive to acute therapy in ED (62.5% of appropriateness), as well as for the hospitalization of preschool children in any case (66.7% of appropriateness), acute-recurrent headaches (66.7% of appropriateness), association with weight increase (50% of appropriateness), mild-to-moderate post-traumatic headache (58.3% of inappropriateness), children requiring non-urgent MRI (58.3% of appropriateness) or other type of non-urgent imaging when CT is normal (66.7% of appropriateness), partial response to therapy in patients without red flags (41.7 to 50% of inappropriateness), or full response to intravenous therapy in status migrainosus (58.3% of appropriateness).

An evident difference in panelists’ opinion was found also in Answer D of Scenario 8, concerning the hospitalization of a patient with headache and general physical examination indicative of acute infection. During the discussion phase, the results were ascribed to a possible misinterpretation of the answer between central nervous system infections, always requiring hospitalization, and other systems infections, potentially not severe.

The indication to discharge the patient from ED after acute symptomatic therapy depends on the presence of red flags and on drug-response, however, the survey results suggested that participants preferred clinical observation also when red flags are absent.

After debate, the final recommendation considered major criteria for hospitalization the age below 5 years, the positivity of neurological examination, an OF showing papilledema or presence of other red flags, even in the case of partial response to therapy.

In the event of a full response to symptomatic therapy of a patient younger than 5 years, the experts decided to underline the importance of a close clinical observation with a possible pediatric neurologist referral within one month.

The OF is a rapid, low-cost procedure requiring a short training, which make it a valid screening test.

Nevertheless, in view of the high rate of untrained personnel among pediatricians and the risk of false positive and false negative findings, the panelists eventually decided to prefer ophthalmologic referral in the case of visual symptoms not clearly related to primary headache or in any case requiring an OF with cycloplegia.

The disagreement about urgent imaging request resulted from scenarios presenting some possible ambiguities. In particular, in Scenario 9, the indication to urgent CT in a case of early-morning headache with vomiting and nausea was rated inappropriate by the 41.7% of participants during the first round and the 62.5% in the second round; appropriateness for hospitalization reached 58.3%. With regards to a case of headache with arterial hypertension and irritability, presented in Scenario 10, an urgent CT was rated more appropriate than a non-urgent MRI (79.2% versus 50% of appropriateness). In addition, the case of Scenario 11, characterized by visual disturbance, vomiting and neck rigidity, the indication to lumbar puncture did not reached agreement (70% of appropriateness).

The controversies were discussed in a second meeting and a general approval of final recommendations was achieved (see Figure 2).

### 4.3. Hospital Admission

Several controversies were found also with the hospital admission phase results. In Scenario 1, the indication to perform MRI for a patient with early-morning headache preceded by vomiting and without other red flags was rated with disagreement, just like the indication for MRA in the case of at least one red flag or post-traumatic headache (included in Scenario 5).

With respect to the association of headache, agitation, confusional status and aphasia, as presented in Scenario 2, the indications for lumbar puncture, EEG and for child neuropshychiatric consultation in order to exclude somatophorm disorders did not reach the agreement, although the rate of appropriateness was high (70.8%, 66.7% and 66.7%, respectively).

Other points of disagreement were found in scenarios 3 and 4, concerning the management of a case with specific visual disturbances and the differential diagnosis between headache and epilepsy.

Most of these controversies referred to specialistic aspects that require a pediatric neurologist support.

Therefore, in final recommendations, the importance of a pediatric neurologist consultation for hospitalized patient was stressed.

### 4.4. Acute Treatment

In acute therapy management, while the indications to first-line therapy were rated with agreement, some discordance derived from the use of anti-emetics and triptans.

In particular, the discouragement of anti-emetics was rated with disagreement (54.2% inappropriate) in Scenario 2, as well as in Scenario 4, in which the use of ondansetron reached a strong agreement in the second round (91.7% of appropriateness).

After meeting discussion, the experts agreed that the use of anti-emetics can be considered in hospital settings in order to favor the use of oral painkiller drugs, although the timely administration of acute therapy is often able to stop also dysautonomic symptoms.

The uncertainties resulted from the first round about the use of triptans (Scenario 3) were resolved after meeting, so that the second round showed agreement in every indication except for triptans efficacy according to the administration route and combination therapy with FANS.

In Scenario 5, only the indication to intravenous hyperhydration for headache patients unresponsive to FANS and triptans was rated with disagreement (66.7% of appropriateness).

These results may be explained by the lower diffusion and manageability in triptans therapy among primary care pediatricians.

### 4.5. Prophylaxis

With reference to prophylactic therapy, the survey results evidenced disagreement on the indications regarding the use of topiramate, valproate, and nutraceuticals, as well as on the general therapeutic management.

In detail, among all of the indications that reached agreement in Scenario 1, 75% of panelists also considered it appropriate to start prophylaxis in the case of responsiveness only for acute polytherapy.

The indication for topiramate and valproate remained uncertain (64.2% and 54.2% of appropriateness, respectively) in Scenario 2, although 83.3% agreed to rate the avoidance of topiramate in the case of anorexia as appropriate in Scenario 3

In addition, agreement was reached (83.3% appropriate) for a therapy duration of 6–12 months in the case of effectiveness, but the decision to withdraw or continue in the case of concurrent onset of adverse effects remained controversial.

The teratogenic risk potential was sufficiently recognized, as demonstrated by the results of Answer B of Scenario 7, although in Scenario 8, the decision about switching a female adolescent on valproate to another form of therapy was rated as appropriate only by 70.8%. Disagreement also resulted for oral contraception dissuasion during prophylactic therapy for migraines, with the relative majority of panelists (62.5%) considering the indication inappropriate (Scenario 7).

The final recommendation obtained after the meeting suggests choosing the drug on the basis of a pediatric neurologist consultation and in relation to comorbidities, adverse events, and patient and parents’ preferences.

While Scenario 5 revealed several points of disagreement with regard to nutraceuticals, biofeedback and drug interaction, accountable for the lack of convincing data in the literature, the broad awareness of the importance of hygiene education was confirmed by general agreement on all of the indications in Scenario 6.

### 4.6. Follow-Up

The follow-up phase required strong collaboration between the primary care pediatrician and pediatric neurologist. From the survey results, we deduced that some aspects of the follow-up monitoring, like the indication for a neurologist reevaluation during topiramate therapy (Scenario 2), were rated with disagreement. Besides, some aspects of home pressure monitoring in patients with headache showed uncertainties among participants. In Scenario 3, indeed, agreement was reached for patients with metabolic syndrome, elevated BMI, and patients on propranolol or amithryptiline therapy, as well as for the importance of parallel monitoring of heart rates, with appropriateness levels ranging from 75% to 95.8% The indications of monitoring every patient with headache, patients with dysautonomic symptoms, or preschoolers remained without agreement. Discussions in the meeting phase highlighted some ambiguities in this scenario, so that panelists, in consideration of the lack of evidence, decided to recommend home pressure monitoring at least in patients on prophylasxis with beta-blockers, calcium antagonists, or amithryptiline.

The survey data demonstrate that training on headache management focused on primary care and hospital pediatricians is essential to improve the healthcare professionals’ network and to quickly detect patients requiring a new specialist assessment. Headaches from medication overuse, for instance, is a topic that pediatricians know little of due to the lack of studies in pediatric populations. We see that, in Scenario 4, the appropriateness rate of diagnostic criteria increased considerably after the meeting.

The RAND proved to be a valid method to value the appropriateness of procedures and define a diagnostic-therapeutic pathway suitable to the local reality. Where points of disagreement remained, expert debate and problem analysis allowed to define a series of shared recommendations.

Among the limitations of this study, it is important to underline the difficulties in summarizing all of the clinical variables involved in pediatric headache in a scenario; however, from the survey’s ambiguities, panelists managed to solve controversies and develop general and inclusive recommendations. To date, there is no convincing method to define the internal and external validity of procedures tested with a RAND/UCLA approach. For validation, our questionnaire was administered twice with a one-week interval to a convenience sample of 10 PCPs and 10 hospital pediatricians. An external validation will begin soon that compares outcomes of patients treated in accordance with the defined recommendations versus patients treated in a different way [8]. Another limitation is the selection of professionals working in a restricted area, whose answers might have been influenced by the organization of the local healthcare system, which potentially not comparable with other national and extranational realities. However, our final recommendations were developed in a general and widely acceptable way and can be used globally in developed countries. The participant drop-out rate between the two rounds is also noteworthy. A possible explanation for this is the lack of incentives to encourage such a large number of panelists to continue the survey.

## 5. Conclusions

Our study provided a schematic pathway for the outpatient and hospital management of pediatric patients with acute and chronic headache. The study also offered specific insights that may help clinicians in differential diagnoses and in specialist consultation referrals, with particular reference to ocular fundus pitfalls, correlations between headache and epilepsy, and psychosomatic disorders. In addition, indications on acute therapy and prophylaxis were developed by integrating the survey results with previous guidelines.

The concurrence of experts’ opinions obtained with a codified consensus measurement, in association with a critical review of the previous literature, allowed to define the appropriateness of procedures and to develop some useful recommendations for optimizing the healthcare professionals’ network related to pediatric headache management. Future studies are still needed to improve data on therapy effectiveness and to clarify the appropriate follow-up approach to each condition.

## Figures and Tables

**Table 1:** Ranking of median and disagreement of expert judgment on scenarios. The judgment was expressed on a scale from 1 to 9, where 1 = definitely inappropriate, 5 = uncertain, 9 = definitely appropriate.

Median	Disagreement	Classification
7–9	No	Appropriate with agreement
7–9	Yes	Appropriate but with disagreement
4–6	Not applicable	Uncertain
1–3	Not applicable	Inappropriate

**Table 2:** Anamnestic data useful in the assessment of children with headache.

**Headache Characteristics**
Onset modalities (sudden onset, progressive)
Duration
Location (frontal, temporal, occipital, parietal)
Quality of pain (pulsating, severe)
Intensity
Premonitory symptoms
Aura
Association with vegetative symptoms (dizziness, sweating, etc.)
Association with neurological symptoms (ictal episodes, walking impairment, speech impairment, diplopia, etc.)
Triggering factors (stress, sleep deprivation, coughing, Valsalva maneuver)
Alleviating factors (rest, staying in the dark)
Efficacy of analgesic therapy
**Proximate pathological history**
Recent trauma
Fever
**Remote pathological history**
Primary headache
Neurocutaneous disorders (e.g., neurofibromatosis)
Congenital heart disease
Immunodeficiency
Neoplasms
Coagulopathies
Peritoneal ventricular shunts
**Family history**
Primary headache
Neurocutaneous disorders
Congenital heart disease
Immunodeficiencies
Coagulopathies
Primary headache
Neurocutaneous disorders

**Table 3:** Red flags in headache. Life-threatening red flags are in italics.

**Headache Characteristics**
*Pain that wakes the child from sleep or occurs on waking*
*Worsening of headache in recumbency and/or during straining, coughing, and/or other forms of Valsalva maneuver, physical activity*
*Sudden severe headache, thunderclap headache*
Recurrent localized headache
Occipital headache
Inadequate response to therapy
*Changes in headache characteristics (both chronic or acute):* intensity, frequency, pattern
*Chronic progressive headache*
**Clinical and medical history**
Incomplete medical and clinical history
Age < 5 y/o
Recent head trauma (<12 h if severe, >12 h if mild)
High-risk population (sickle cell anemia, coagulopathy, immunodeficiency, former or present tumor, cardiac diseases with right-left shunt, hydrocephalus or ventricular shunt, type 1 neurofibromatosis, tuberous sclerosis, familiar genetic syndromes)
Changes in mood, behavior, or personality *
*Polyuria, polydipsia*
*Morning/fasting nausea and/or vomiting*
Growth anomalies (head circumference increase, short stature or growth reduction, precocious/delayed puberty)
Neck/rachis symptoms
Systemic disease symptoms (weight loss, night sweats, fever, joint pain)
**Clinical data from physical examination**
Reduced general condition
*Neurological signs and symptoms (persistent nausea and/or vomiting, impaired mental state, ataxia, asymmetric, weakness, focal deficits)*
*Motor signs and symptoms (regression of psychomotor development, focal deficits, gait abnormalities, impaired coordination, impaired swallowing)*
*Visual disorders (papilledema, retinal hemorrhage, pathological pupillary response, diplopia, nystagmus or abnormal ocular movements, visual field defects, visual acuity defect reduction unrelated to abnormal ocular refraction)*
Incompliant child (impossibility to perform physical examination)
Neck stiffness or other meningeal signs
Trauma
Cranial vascular murmur
Neurocutaneous markers
Macrocephaly

* In children, changes in mood and behavior might only indicate worsening pain.

**Table 4:** Headache temporal patterns.

**Acute Headache**	**Recurrent Acute Headache**
Upper respiratory tract infections with or without fever	Migraine Tension headache
Acute sinusitis	Cluster headache
Pharyngitis	Hypertension
Viral or bacterial meningitis	Hyperhyroidism
Migraine (first episode)	Pheocromocytoma
Drug abuse	Drugs
Poisoning	
Medications (steroids, oral contraceptives)	
Ventricular-peritoneal shunt malfunction	
Brain tumor	
Subarachnoid haemorrhage	
Intracranic haemorrhage	
Hydrocephalus	
Venous sinuses thrombosis	
**Chronic progressive headache**	**Non progressive chronic headache**
Brain tumor	Chronic migraine
Communicating or obstructive hydrocephalus	Tension-type headache
Pseudotumor cerebri	Cluster headache
Cerebral abscess Subdural chronic hematoma	
Aneurysms and vascular malformations Drugs	
Poisoning	
**Daily persistent new onset headache**	
Primary acute headache and chronic headache association	

**Table 5:** Full objective examination of headache patients.

**General and Vital Signs Assessment**
General condition (complexion, hydration status)
Skin temperature
Cardiorespiratory activity
Blood pressure
Auxological parameters: weight, height, head circumference
**Physical examination**
Skin: search for skin discolorations
Examination of algogenic structures of the head and neck: sinuses, temporomandibular joints, identification of trigger points, pain points or muscle contractures
Palpation of the thyroid gland
Assessment of nuchal stiffness
Pubertal state
**Systematic neurological examination**
Mental and psychic status (drowsiness, irritability), alertness, responsiveness, speech
Asymmetries and side marks
Ocular system: pupillary reflexes, extrinsic ocular motility with nystagmus and diplopia search, visual field
Fundus oculi
Cranial nerves: facial symmetry, strength and sensitivity of facial districts, signs of focal pathology
Marching, balance, coordination, basic gait planting
Strength and sensitivity
Osteotendinous reflexes (in the infant, archaic reflexes)

**Table 6:** General characteristics of primary and secondary headaches.

	Primary Headache	Secondary Headache
**Duration of disease**	Chronic, >6 months	Acute-subacute
**Temporal pattern**	Recurring or daily	Progressive
**Localization**	Frontal, temporal	Occipital
**Features**	Pulsating, squeezing	Gravitating
**Intensity**	Mild to severe	Generally severe
**Time of the day**	Indifferent	Upon awakening in the morning
**Episode duration**	Hours to days	Continuous
**Neurological objectivity**	Normal	Impaired
**Emetic symptomatology**	Nausea > vomit	Vomit > nausea
**Visual symptoms**	Visual aura	Diplopia
**Phonophobia and photophobia**	Possible	Absent

**Table 7:** Triptans and associated indications for pediatric use.

Drug	Dose	Age Indications	Notes
ALMOTRIPTAN	6.25–12.5 mg tabs	≥12 years old	Efficacy on phonophobia and photophobia
RIZATRIPTAN	5–10 mg OSF	≥5 years old	Reduce dose in the case of simultaneous use of propranolol
SUMATRIPTAN	5–20 mg NS	≥12 years old	Efficacy on phonophobia and photophobia
ZOLMITRIPTAN	5 mg NS	≥5 years old	Efficacy at lower doses compared to other triptans

Tabs = tablets; OSF = orosoluble formulation; NS = nasal spray.

**Table 8:** Second-line combination therapy in adolescents with an unsatisfactory response to triptans.

Drug	Dose	Notes
TRIPTAN + IBUPROFEN		Multiple formulations available
SUMATRIPTAN + NAPROXEN	10/60 mg Tabs 30/180 mg Tabs 85/500 mg Tabs	Efficacy regarding phono- and photophobia Efficacy demonstrated even at the lowest dose Minimum age 12 years

Tabs = tablets.

**Table 9:** Antiemetic drugs used for management of vomiting during an episode of headache.

Drug	Dose	Notes
ONDANSETRON	OS, repeatable every 8 h: If <15 kg: 0.2 mg/kg If 15–30 kg: 4 mg If >30 kg: 4–8 mg	Sedation Dystonias
PROCHLORPERAZINE	OS: If 10–13 kg: 2.5 mg every 12–24 ore If 13–18 kg: 2.5 mg every 8–12 ore (maximum 10 mg/day) If 18–40 kg: 2.5 mg every 8 h or 5 mg every 12 h IV: 0.1–0.15 mg/kg/dose	Sedation Dystonias
PROMETHAZINE	OS, R: 0.25–1 mg every 4–6 h	Sedation Dystonias

OS = oral; IV = intravenous; R = rectal.

**Table 10:** Second-line intravenous therapy as an alternative to NSAIDs in acute headache unresponsive to first-line therapy.

Drug	Dose	Maximum Dose
INTRAVENOUS HYDRATION + PROCHLORPERAZINE + IV KETOROLAC	0.15 mg/kg 0.5 mg/kg	10 mg 30 mg
SUMATRIPTAN	3–6 mg NS	20 mg in 24 h
METOCLOPRAMIDE	0.2 mg/kg IV	10 mg
DIHYDROERGOTAMINE (DHE)	0.5 mg in 3 min IV	

IV = intravenous; NS = nasal spray.

**Table 11:** Drugs for preventive pediatric headache treatment.

Drug	Dose	Maximum Dose	Notes
AMITRIPTYLINE (PLUS CBT)	Initial dose: 0.25–0.50 mg/kg/day To be increased in the case of ineffectiveness Maintenance: 1 mg/kg/day	75 mg/day	Side effects: suicidal ideation. For patients aged 12 years and older with depression or anxiety disorders.
TOPIRAMATE	Initial dose: 1–2 mg/kg/day (in younger than 12 years); 2.5 mg/kg/day (in aged 12 years and olders) for a week	100 mg/day	Side effects: teratogenicity (caution in women of childbearing age).
VALPROATE	Initial dose: 10–15 mg/kg/day in 2 doses	30 mg/kg/day	Side effects: teratogenicity (caution in women of childbearing age).
CINNARIZINE	1.5 mg/kg/day if weight <30 kg 50 mg/day if weight >30 kg		Contraindicated in patients younger than 12 years.
FLUNARIZINE	3–5 mg/day in a single daily dose	10 mg/day	Side effects: weight gain, sedation. Contraindicated in patients younger than 12 years.
NIMODIPINE	10–20 mg/day in 3 doses	150 mg/day	Side effects: mild gastrointestinal disturbances. Contraindicated in patients younger than 12 years.
PROPRANOLOL	Initial dose: 1 mg/kg/day in 3 doses Maintenance: 20–40 mg/day in 3 doses	3 mg/kg/day	Use with caution in asthmatic, depressed or diabetic patients.

CBT: cognitive-behavioural therapy.

**Table 12:** Main nutraceuticals used in pediatric age.

Nutraceutical	Dose	Notes
5-HYDROXYTRYPTOPHAN	5 mg/kg/day	Side effects: nausea, abdominal pain, drowsiness
MAGNESIUM	1.5–4.5 g/day	Side effects: flatulence, diarrhea. Contraindications: severe renal failure
RIBOFLAVIN	25–400 mg/day	Side effects: yellowish urine, gastro-intestinal symptoms
MELATONIN	3 mg/day before night sleep	High doses may cause daytime drowsiness, impaired physical and mental performance, suppressed body temperature and elevated blood prolactin levels

**Table 13:** Consensus recommendations list.

**Primary Care Pediatrician Evaluation**
**Recommendation 1**: In the case of acute headache in a pediatric patient, the PCP should identify the red flags that require urgent diagnostic and therapeutic procedures by an adequate history collection, in order to distinguish primary from secondary forms.
**Recommendation 2**: Assessment of vital signs, general physical examination, and a complete neurological examination should always be performed in a child with headache, as this can identify warning signs suggestive of secondary headache. In particular, the presence of high-risk red flags suggestive of severe conditions such as infectious processes, vascular lesions or intracranial expansions should be excluded. The neurological examination should include: assessment of level of consciousness, meningeal signs, visual, gait and co-ordination disturbances, speech and hearing disorders, focal neurological deficits such as localized strength or sensory deficits, cranial nerve deficits. The OF is a useful non-invasive examination that a pediatrician can perform if sufficiently experienced. In doubtful cases, an ophthalmologist evaluation for fundoscopy with pupil dilation is recommended.
**Recommendation 3:** The role of the PCP is to detect potentially dangerous or life-threatening conditions that require an immediate emergency setting medical evaluation. In the case of neurological alterations at physical examination, or in the presence of other warning signs, the patient should be immediately addressed to the ED. In the case of a general physical examination suggestive of secondary and non-life-threatening conditions, the underlying causes should be treated and an initial analgesic therapy approach should be performed under close monitoring, especially in preschool patients. An ED referral is recommended only in the case of general worsening, therapy failure, appearance of warning signs/symptoms, or positive neurological examination.
**Recommendation 4:** In the case of normal general and neurological examination, without red flags, after investigating the patient’s environmental context and presence of trigger factors, the PCP may recommend acute antalgic therapy, the use of a headache diary, and plan a clinical re-evaluation to assess symptoms evolution and response to therapy. Referral to the pediatric neurologist is recommended in the following cases: children under 5 years of age, lack of or insufficient response to therapy, worsening of symptoms, or in general, for a specialist diagnostic definition.
**Emergency department evaluation**
**Recommendation 5:** During triage, patients not requiring urgent care should be distinguished from those with red flags suggestive of life-threatening disease. In ED, laboratory tests are indicated in the case of a positive general physical examination for secondary forms or when a benign form (e.g., infectious) is suspected, in order to define etiological therapy. Ophthalmologic consultation should be considered whenever a visual symptom is identified and is not clearly correlated with primary headache or in all cases that require an ocular fundus in cycloplegia. Urgent consultation with a pediatric neurologist is indicated, whenever possible, in cases presenting neurological signs or symptoms; it may be deferred to an elective consultation in cases of headache symptoms suggestive of primary or secondary non-life-threatening forms that can be treated with symptomatic therapy.
**Recommendation 6:** Hospitalization is indicated for patients who, irrespective of age, present with a positive general physical examination for benign secondary forms whose causes require further diagnostic and therapeutic investigation and/or alterations on neurological examination and/or fundus oculi indicative of papilledema and/or red flags. In preschool patients (<5 years old), it may be appropriate to hospitalize even forms with negative neurological and general examination but absent response to symptomatic therapy. A short observation regimen may be planned for similar cases in school-aged patients (>5 years).
**Recommendation 7:** CT scan without contrast medium is the test of choice in the emergency setting, due to its rapidity of execution and its easier accessibility in the ED, without the need for patient sedation. In the case of suspected stroke, brain MRI should be performed within one hour of patient arrival in the ED.
**Hospital admission**
**Recommendation 8:** During hospitalization, a close monitoring of parameters and clinical conditions is appropriate to assess response to therapy and possible appearance of “red flags” for secondary headaches. The choice of diagnostic investigations depends on clinical suspicion and is supported by the evaluation of the pediatric neurologist. In the case of a positive general examination, it is recommended to perform microbiological and specific instrumental examinations to exclude infections. The appearance of neurological warning signs during hospitalization is an indication to perform urgent neuroimaging to exclude life-threatening conditions.
**Recommendation 9:** In the differential diagnosis between primary headache and epilepsy, it is useful to consider some characteristic clinical features and to consult a pediatric neurologist who may request an EEG.
**Recommendation 10:** Red flags identified through the collection of anamnestic data, general and neurological physical examination, should direct to in-depth examinations through neuroimaging. In an inpatient setting, MRI is preferred in the suspicion of lesions of the saddle, craniocervical junction, posterior fossa, white matter abnormalities, congenital malformations and infectious processes, as it is more sensitive than CT and safer in terms of radiation protection. For the evaluation of ischemic events or vascular malformations, it is recommended to consider in addition to the standard sequences also CT angiography or MRA depending on availability and level of urgency.
**Acute treatment**
**Recommendation 11:** In the management of acute headache attack in pediatric patient with no definite diagnosis, ibuprofen is a first-line therapy for his rapidity of action. As an alternative, it is possible to use paracetamol, the efficacy of which is similar to that of ibuprofen, despite lower rapidity. The preferred route of administration is the oral one, using buccal formulations.
**Recommendation 12:** In the case of acute attack in a patient with diagnosis of migraine, in order to treat associated symptoms (phonophobia, photophobia, nausea and vomiting), physicians should explain to the patient that they regress with analgesics taken at the beginning or when pain is still low to moderate. The oral way of administration is the advisable one, in buccal formulations. It is possible to associate an antiemetic treatment in order to promote oral therapy; if nausea and vomiting make the oral route difficult, it may be necessary to refer the patient to the nearest ED to gain a venous access and administer fluids, analgesic and antiemetic therapy.
**Recommendation 13:** Triptans are indicated in adolescents with migraine, possibly in association to ibuprofen in the case of insufficient response to monotherapy. Triptans approved in pediatric age are four: almotriptan, sumatriptan, zolmitriptan, for children older than 12 years, and rizatriptan, for children older than 6 years. Off-label use for younger ages is possible under pediatric neurologist prescription. Their use is restricted to acute pain not responding sufficiently to treatment with non-steroidal anti-inflammatory drugs and/or paracetamol and they should be taken when pain is still low to moderate for a rapid and safe response, even though they may be still effective in cases of pain from moderate to severe. They are available in oral and nasal formulations. Both way of administration showed a similar efficacy, even though the oral one is better tolerated.
**Recommendation 14:** Even though analgesic treatment alone is shown to control associated symptoms, the administration of antiemetic drugs can be considered with a double effect of limiting nausea and vomiting, often associated with headache, and promote oral administration of analgesics, when necessary. The main drugs approved in these conditions are ondansetron, serotonin receptors antagonist, used in hospital, and dopamine antagonists such as promethazine and proclorperazine.
**Recommendation 15**: In children presenting with acute migraine attack, not responding to first line treatment, or status migrainosus, a venous access should be established to start intravenous hyperhydration and intravenous paracetamol or NSAIDs (ibuprofen or ketorolac) therapy. Metoclopramide is a dopamine antagonist alternative to first line analgesics, that showed efficacy in treating both vomiting and pain. In the case of status migrainosus, the therapy consists in intravenous administration of dihydroergotamine at high (0.5–1 mg/kg every 8 h) or low (0.1–0.2 mg/kg every 8 h) dose.
**Prophylaxis**
**Recommendation 16**: The indications for preventive therapy in pediatric headache are: attacks of high intensity or occurring with high frequency (more than 4 days/month with 3–4 headache attacks/month for at least three months), reduction in patient’s quality of life, poor responsiveness to acute treatment with disability. The decision to undertake preventive therapy is a responsibility of the pediatric neurology specialist. Prophylaxis may also prevent adverse events of drug abuse, such as medication overuse headache.
**Recommendation 17**: The current guidelines only provide the use of topiramate, propranolol, amitriptyline + CBT and cinnarizine for the prophylactic treatment of migraine, for which the analysis of the evidence has given satisfactory results in terms of efficacy.
**Recommendation 18**: To date, efficacy data of the main drugs used for headache prophylaxis are controversial. The choice of the appropriate drug is based on comorbidities, adverse effects and parents’ and patient’s preferences.
**Recommendation 19**: Preventive drug therapy should be started at the lowest possible doses. Doses should then be gradually increased over 3–4 weeks until therapeutic benefit develops, the maximum administered dose is reached, or significant side effects occur. Treatment should be continued for 7–8 weeks at the correct effective dose to achieve real benefit. If migraine episodes decrease in frequency and intensity, therapy should be continued for approximately 6–12 months.
**Recommendation 20**: The use of nutraceuticals in the preventive treatment of headache remains debated. In clinical practice, nutraceuticals are not contraindicated in mild primary headaches or in support of drug therapy, considering their good tolerability and safety.
**Recommendation 21**: Physicians should explain to patients and families that lifestyle and certain behavioral factors can influence the frequency of headache and should educate patients and their families to identify and change factors that act as triggers for headache.
**Recommendation 22:** The use of complementary therapies for headache prevention is a possible alternative to pharmacological prophylaxis. In certain patients, on the advice of the specialist, it may be considered an effective first-line therapy. Complementary therapies may also be useful in situations of poor patient and parental compliance, poor tolerance and/or presence of contraindications to drug therapy.
**Follow-up**
**Recommendation 23**: A specialist re-assessment should be recommended every time a patient taking prophylactic therapy shows significant adverse reactions, inadequate tolerance to the drug or rapid changes in the headache pattern during the first months.
**Recommendation 24:** Physical examination of a patient with headache must include heart rate and blood pressure assessment. Evidence from the literature do not give precise indications on blood pressure monitoring in patients with headache but it is considered mandatory in patients assuming beta blockers, calcium channel blockers and amitriptyline.
**Recommendation 25**: Medication overuse headache is a concrete risk in the pediatric age. The PCP must monitor acute treatment in patients with headache. Assumption of NSAIDs for more than 14 days/month, triptans/ergotamine for more than 9 days/months, combined drugs for more than 3 months are criteria for a specialist re-evaluation and start of a preventive therapy.

**Table 14:** Roles of the physicians involved in the headache healthcare network.

Primary Care Physicians	Hospital Physicians	Pediatric Neurologists
Evaluate non-critical patients with headache	Detect red-flags requiring urgent imaging and hospital admission	Specialist management of primary and secondary headache patients
Detect red-flags requiring immediate emergency setting medical evaluation	Detect underling conditions requiring specific treatments	Support primary care and hospital physicians for diagnosis and treatment
Treat non-life-threatening headache and underlining conditions	Treat and observe patients not-responding to oral acute therapy	Follow-up
Monitor specific cases and require specialist consultations when indicated	Request specialist consultation when indicated	

## Data Availability

All the data are included in the manuscript and the Supplementary Material.

## Supplementary Materials

The following supporting information can be downloaded at: https://www.mdpi.com/article/10.3390/life12020142/s1, Material S1 includes the questionnaire, Material S2 is represented by answers to the surveys.
